# Supplementary figures and images for: Single nucleotide polymorphisms and copy-number variations in the Trypanosoma brucei repeat (TBR) sequence can be used to enhance amplification and genotyping of Trypanozoon strains
Source: PLoS One. 2021 Oct 25;16(10):e0258711. doi: 10.1371/journal.pone.0258711 (PMC8544829; doi:10.1371/journal.pone.0258711)

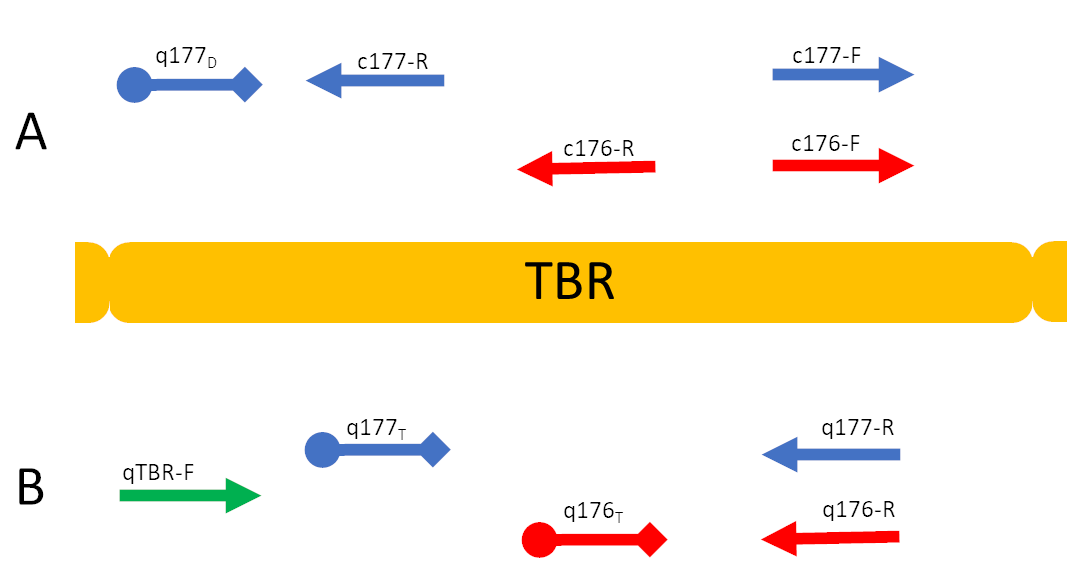

Supplement: S1 Fig — A representation of a TBR sequence as tandem repeat (yellow) showing the relative position of primers and probes used in conventional and quantitative PCR. Arrows indicate the 5’– 3’ direction of primers, while for probes, circles indicate fluorophores and diamonds indicate quenchers. In A, blue indicates the c177 and the q177D set, while red represents the c176 set. In B, the green arrow represents the common primer qTBR-F, while the blue primer and probe indicate the q177T set, and the red primer and probe indicate the q176T set. (TIF) [file pone.0258711.s001.tif]

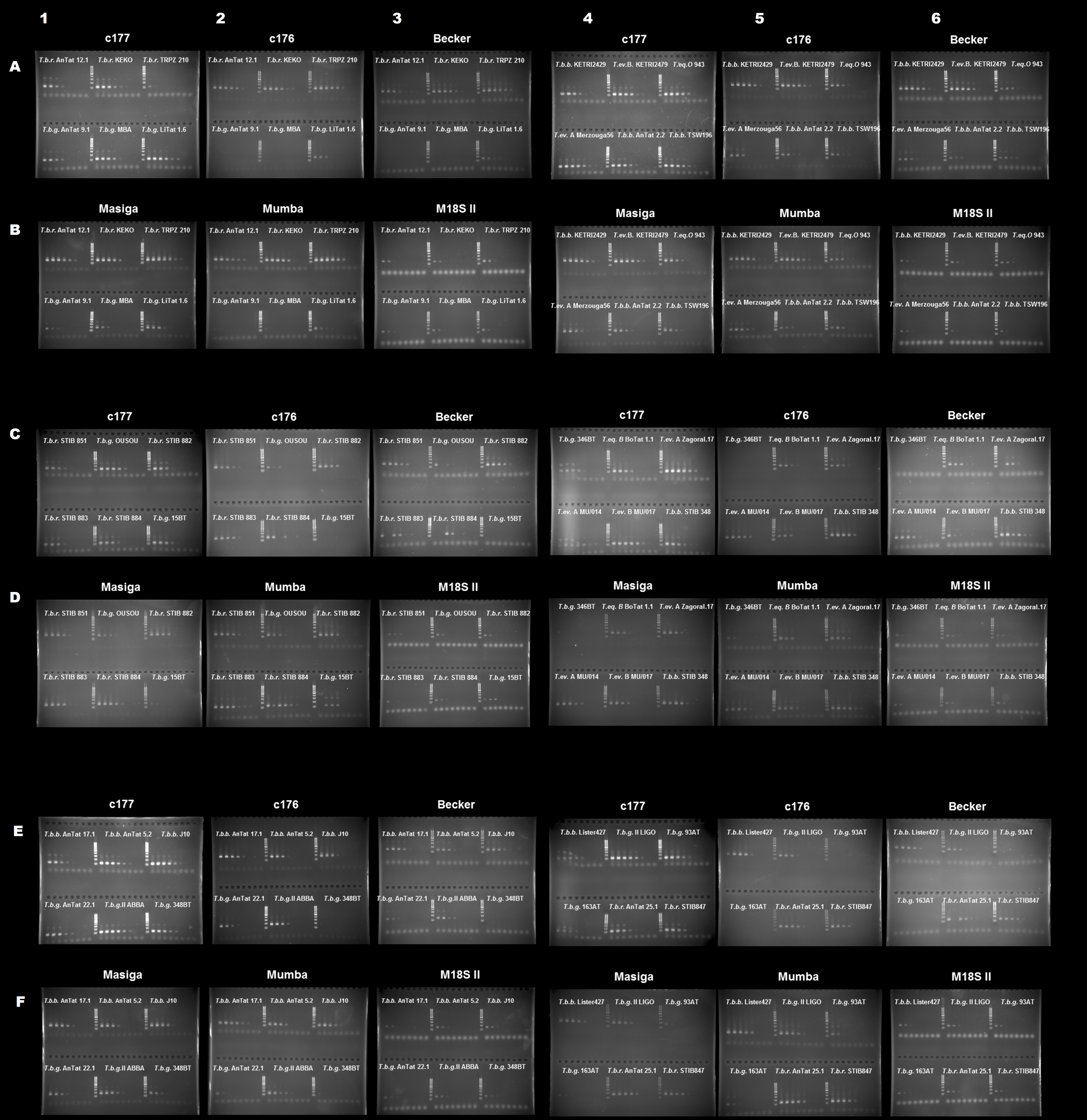

Supplement: S2 Fig — Semi-quantitative conventional PCRs using the Masiga, Becker, Mumba, c177, c176 and M18S II primer sets on fivefold serial dilutions containing 2000, 400, 80, 16, 3.2, 0.64, 0.128 or 0 fg of pure parasite DNA per lane. In total, 36 Trypanozoon strains were tested. Each of the gels shows the electrophoretic results of one of the conventional PCRs tested on 6 Trypanozoon strains (3 above and 3 below), separated by 5 μl of the Generuler 100-bp DNA ladder (Thermo Scientific). (TIF) [file pone.0258711.s002.tif]

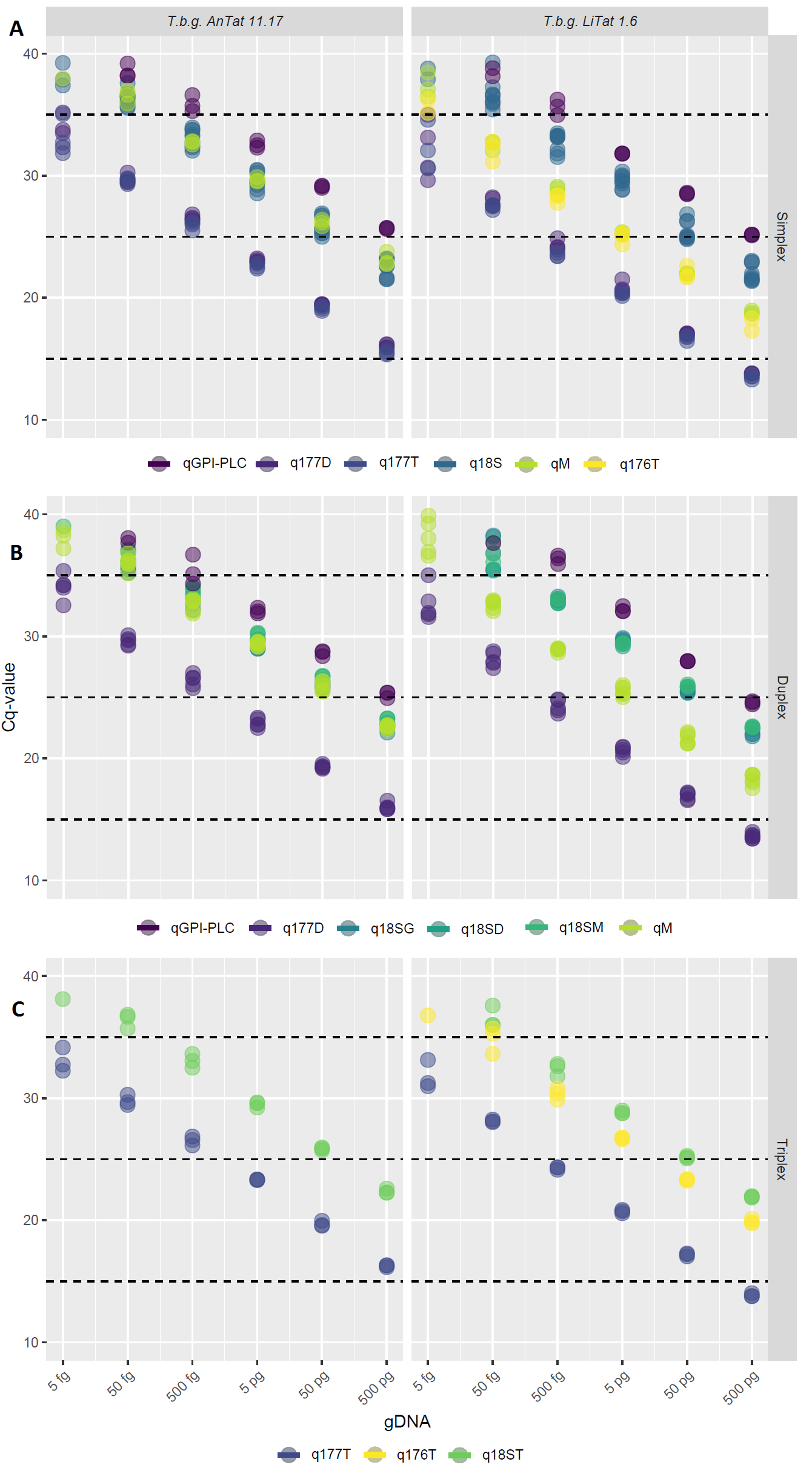

Supplement: S3 Fig — Cq-values obtained from a tenfold dilution series from 500 pg down to 5 fg of pure parasite DNA (in elution buffer) of two T.b.g. I clones: AnTat 11.17 and LiTat 1.6 in different qPCR formats: simplex, duplex (in combination with q18S) or triplex (as qTBR). The slope of qPCR efficiency was estimated by fitting a linear trendline on Cq values and the log transformed concentrations. (TIF) [file pone.0258711.s003.tif]

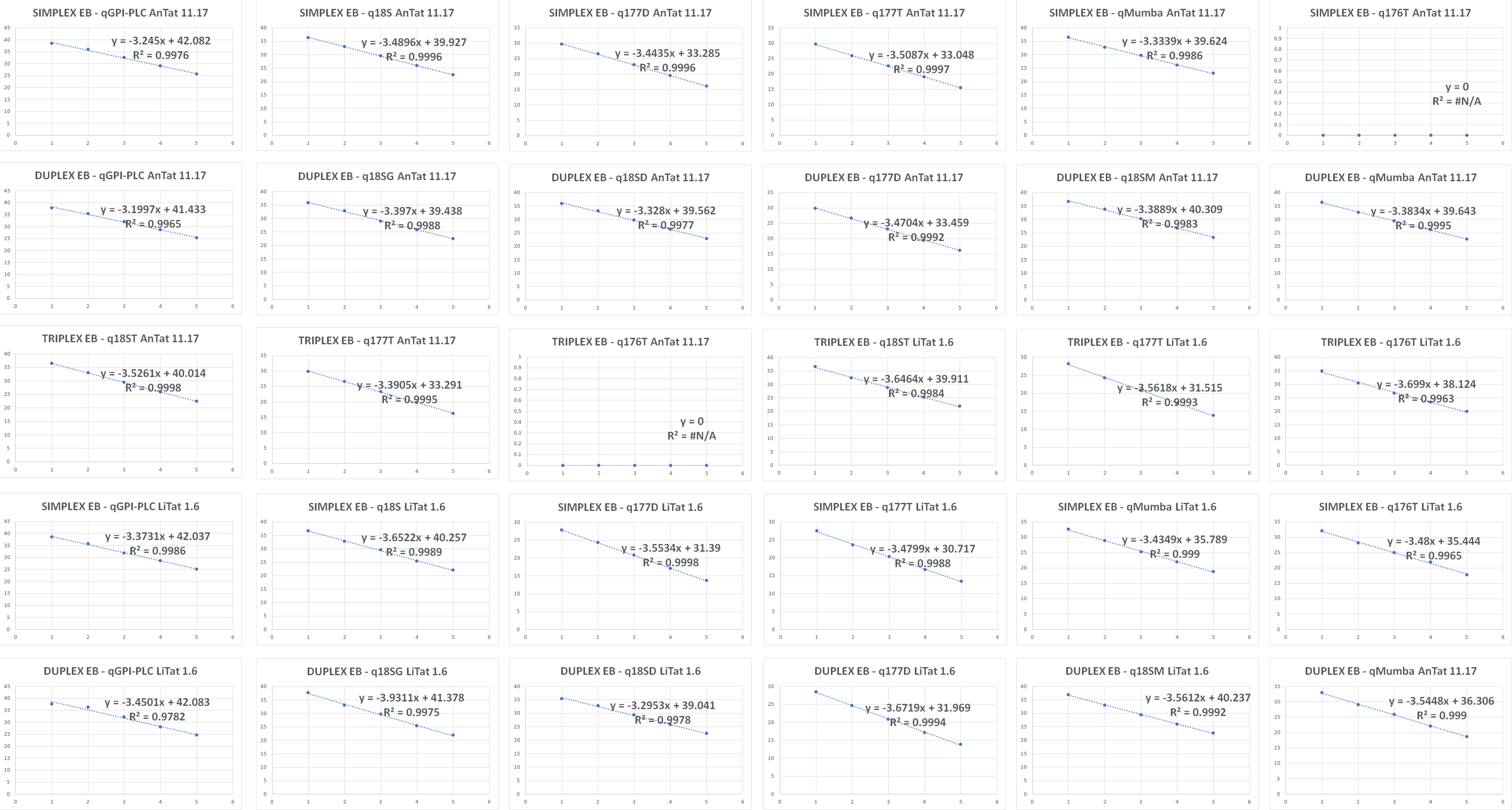

Supplement: S4 Fig — Cq-values obtained from a tenfold dilution series from 500 pg down to 5 fg of pure parasite DNA (in elution buffer) of two T.b.g. I clones: AnTat 11.17 and LiTat 1.6 in different qPCR formats. Each of the novel qPCRs was first tested individually in simplex format (A). qGPI-PLC, q177D or qM, was combined with q18S in duplex format (B). q177T and q176T were combined with q18S in triplex format, representing the qTBR (C). (TIF) [file pone.0258711.s004.tif]

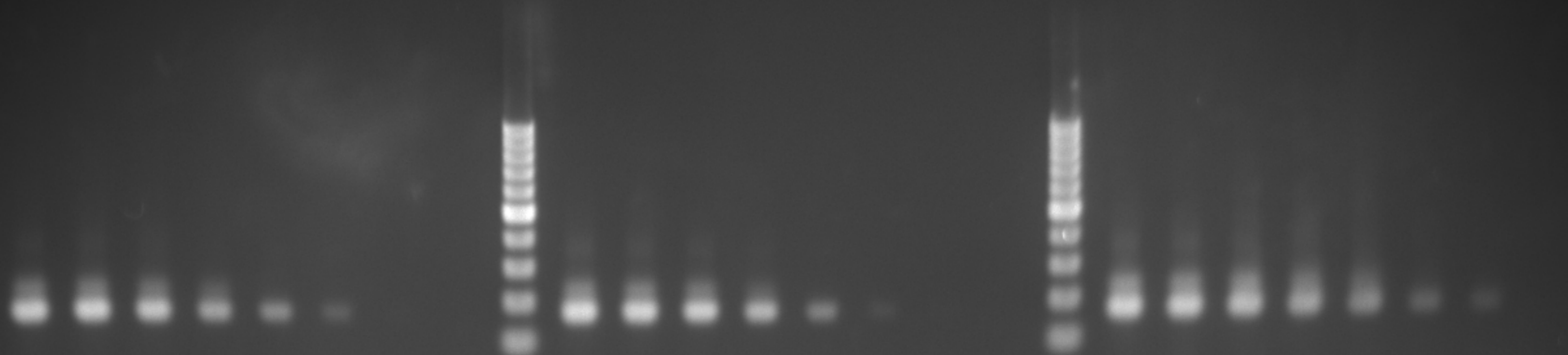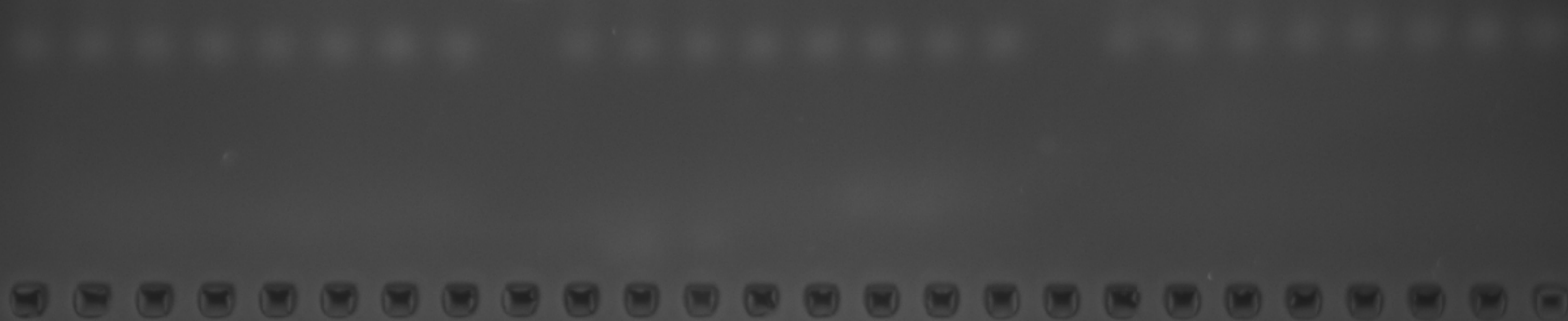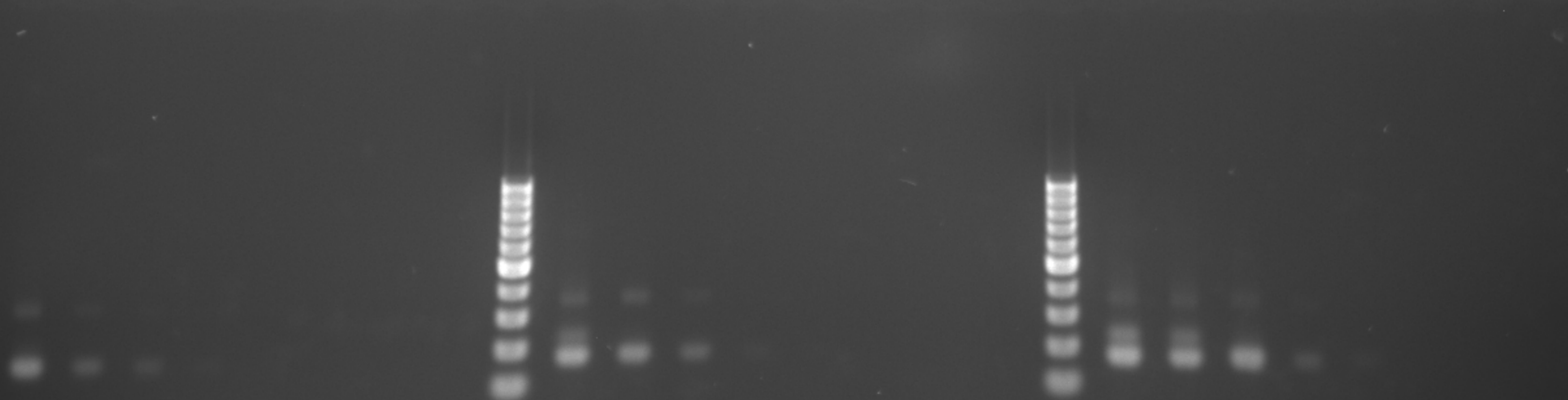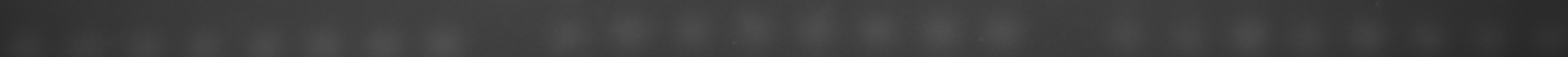

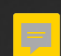

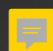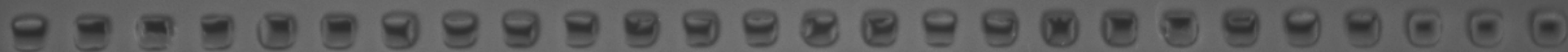

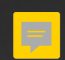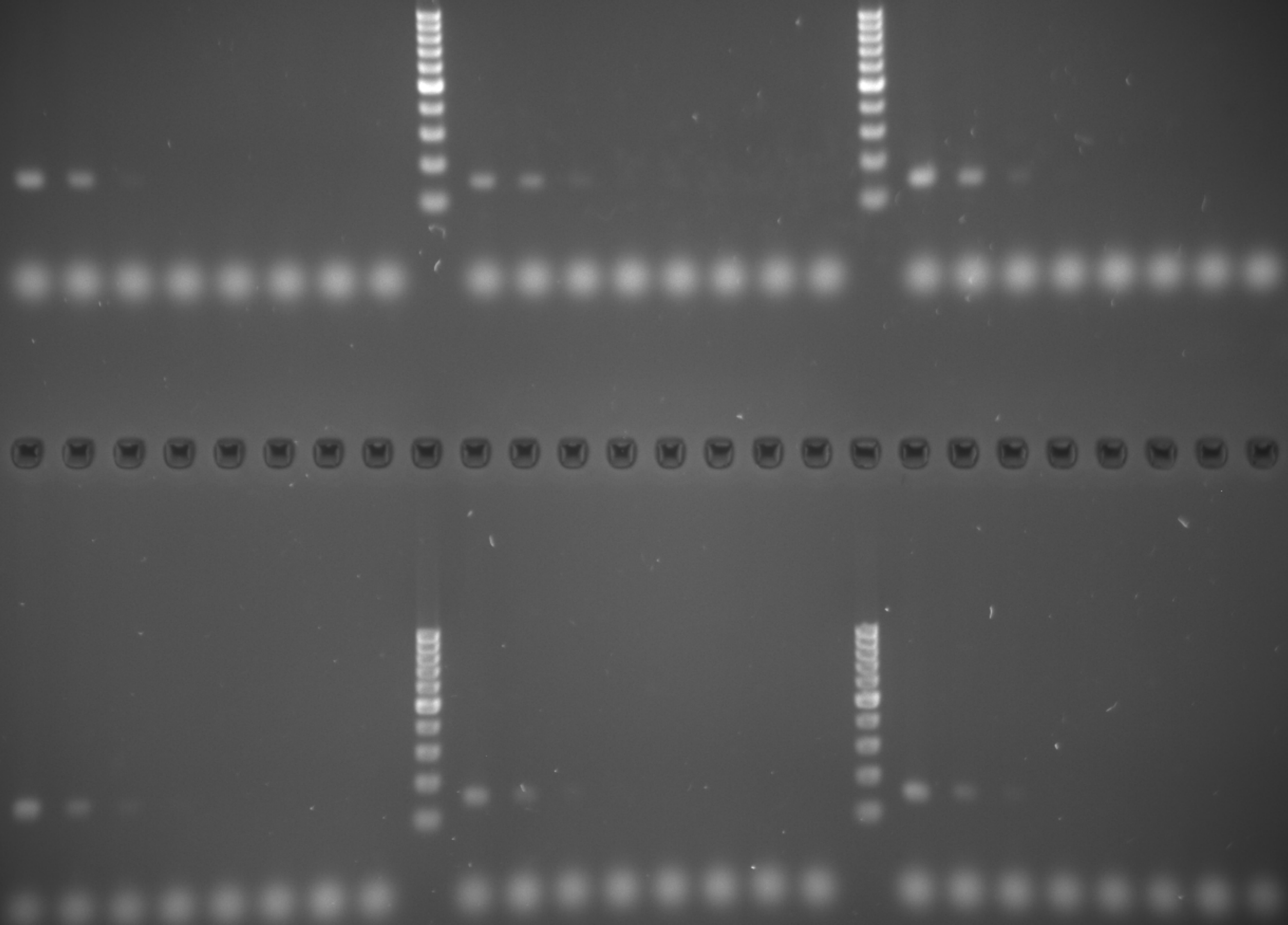

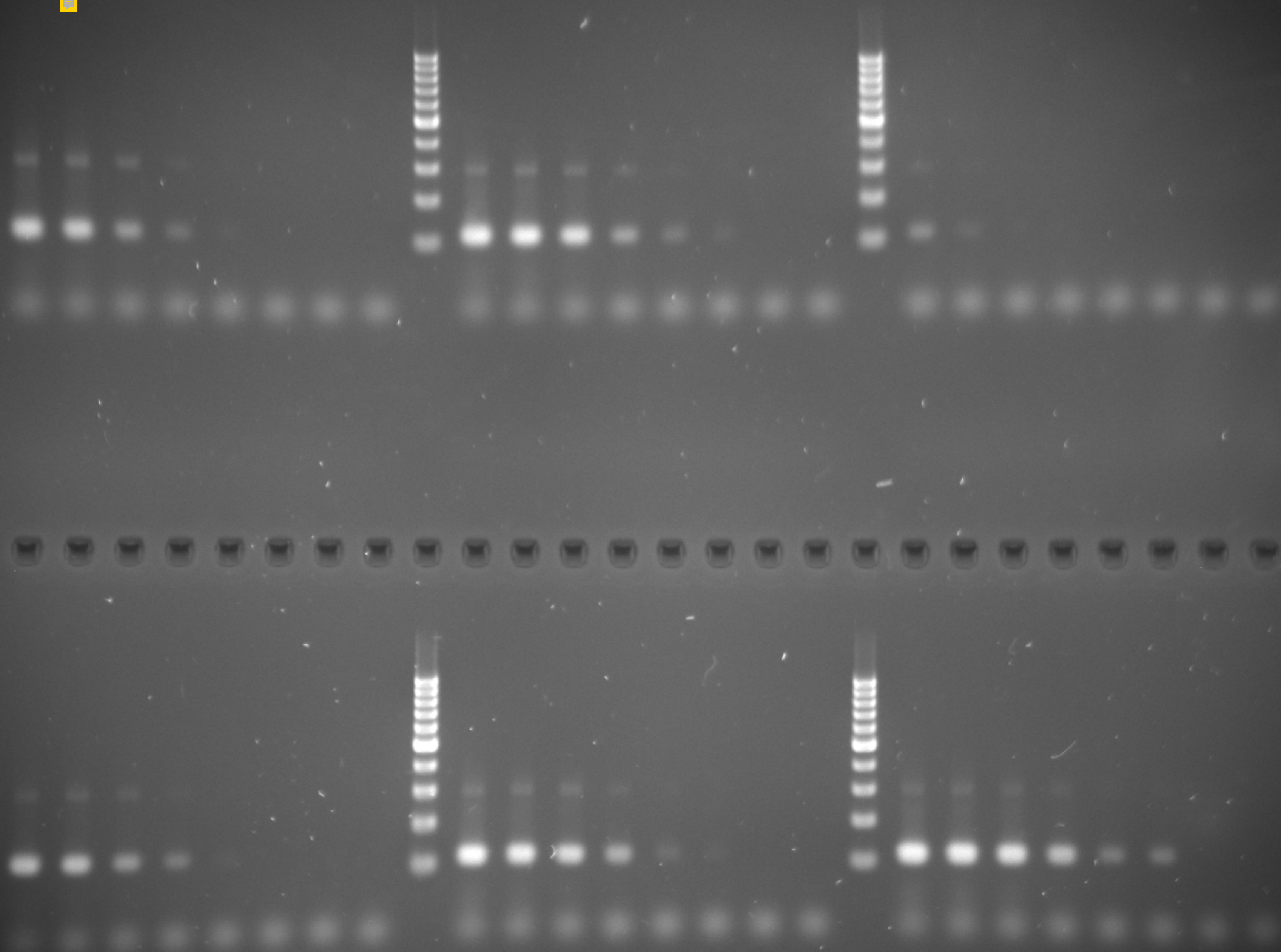

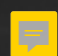

Supplement: S1 Raw images — (PDF) [file pone.0258711.s006.pdf]
