# Supplementary material for: Single nucleotide polymorphisms and copy-number variations in the Trypanosoma brucei repeat (TBR) sequence can be used to enhance amplification and genotyping of Trypanozoon strains
Source: PLoS One. 2021 Oct 25;16(10):e0258711. doi: 10.1371/journal.pone.0258711 (PMC8544829; doi:10.1371/journal.pone.0258711)

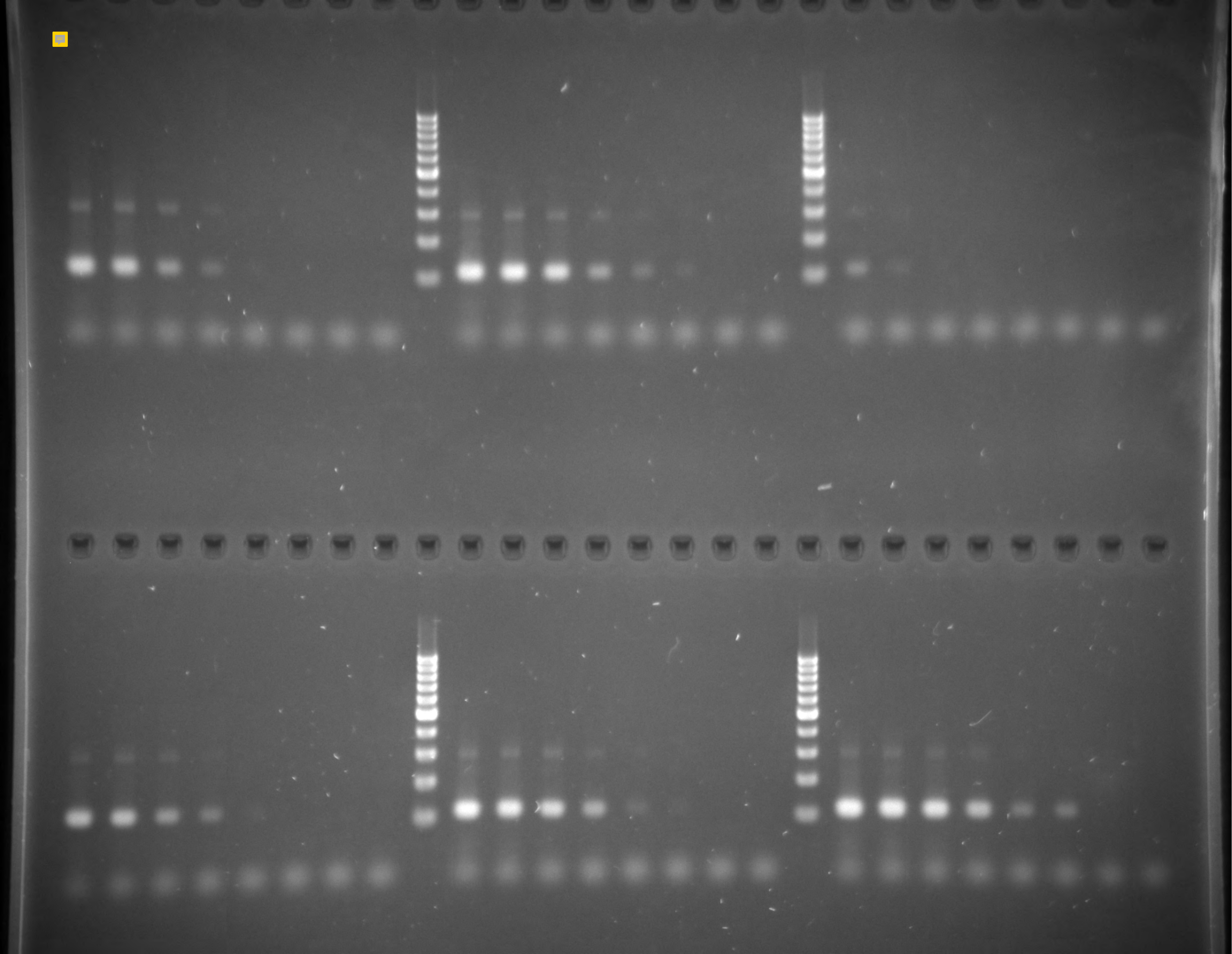

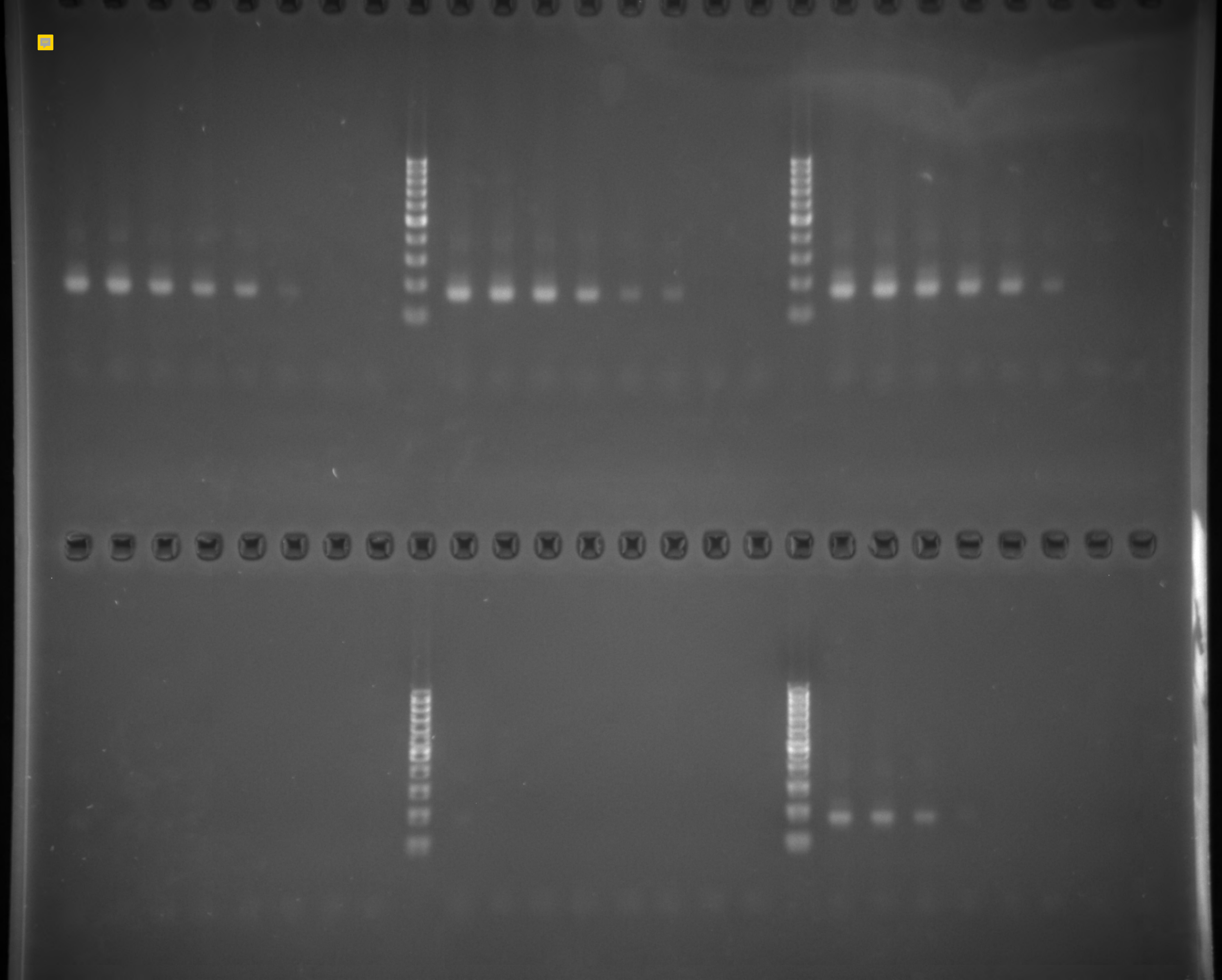

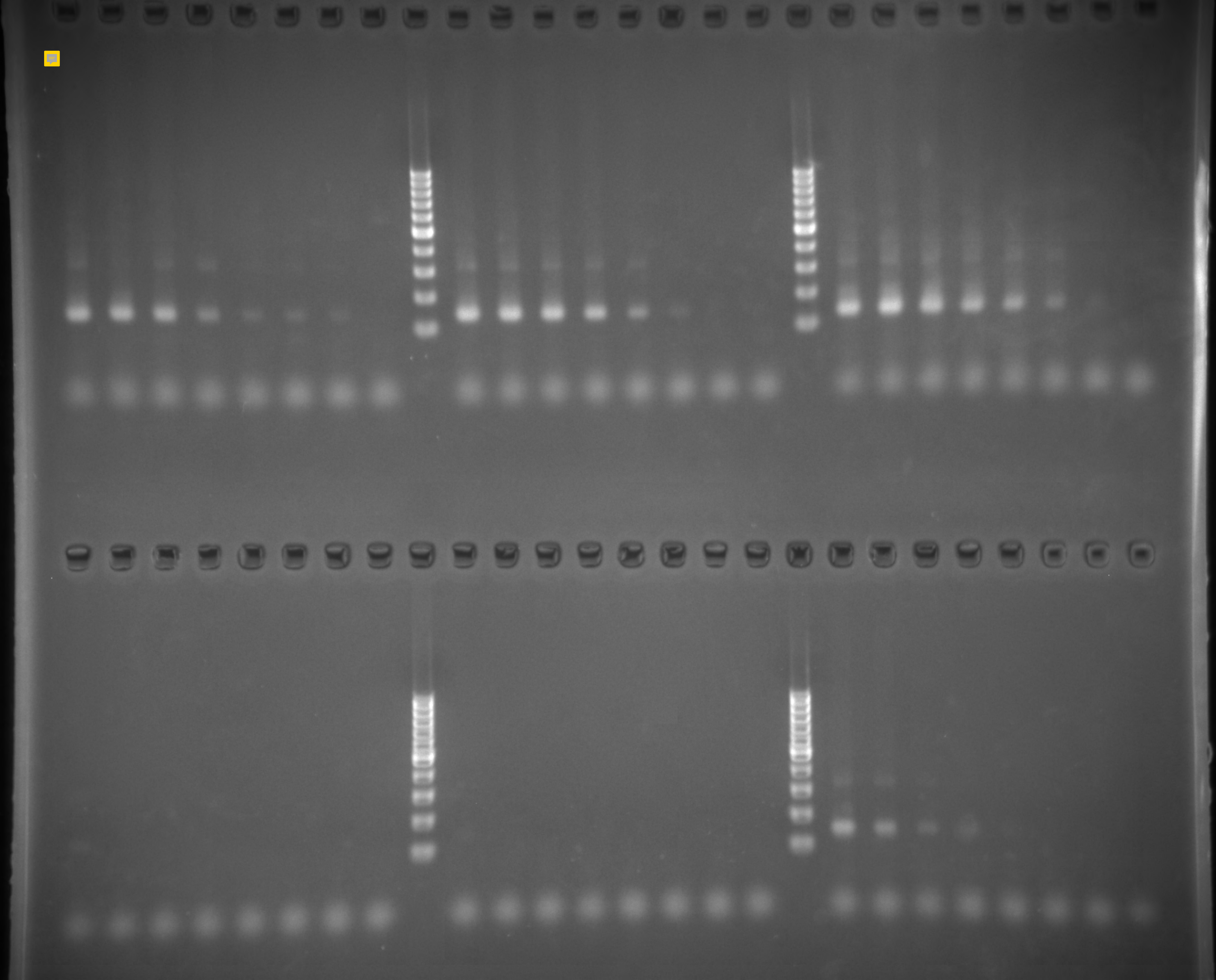

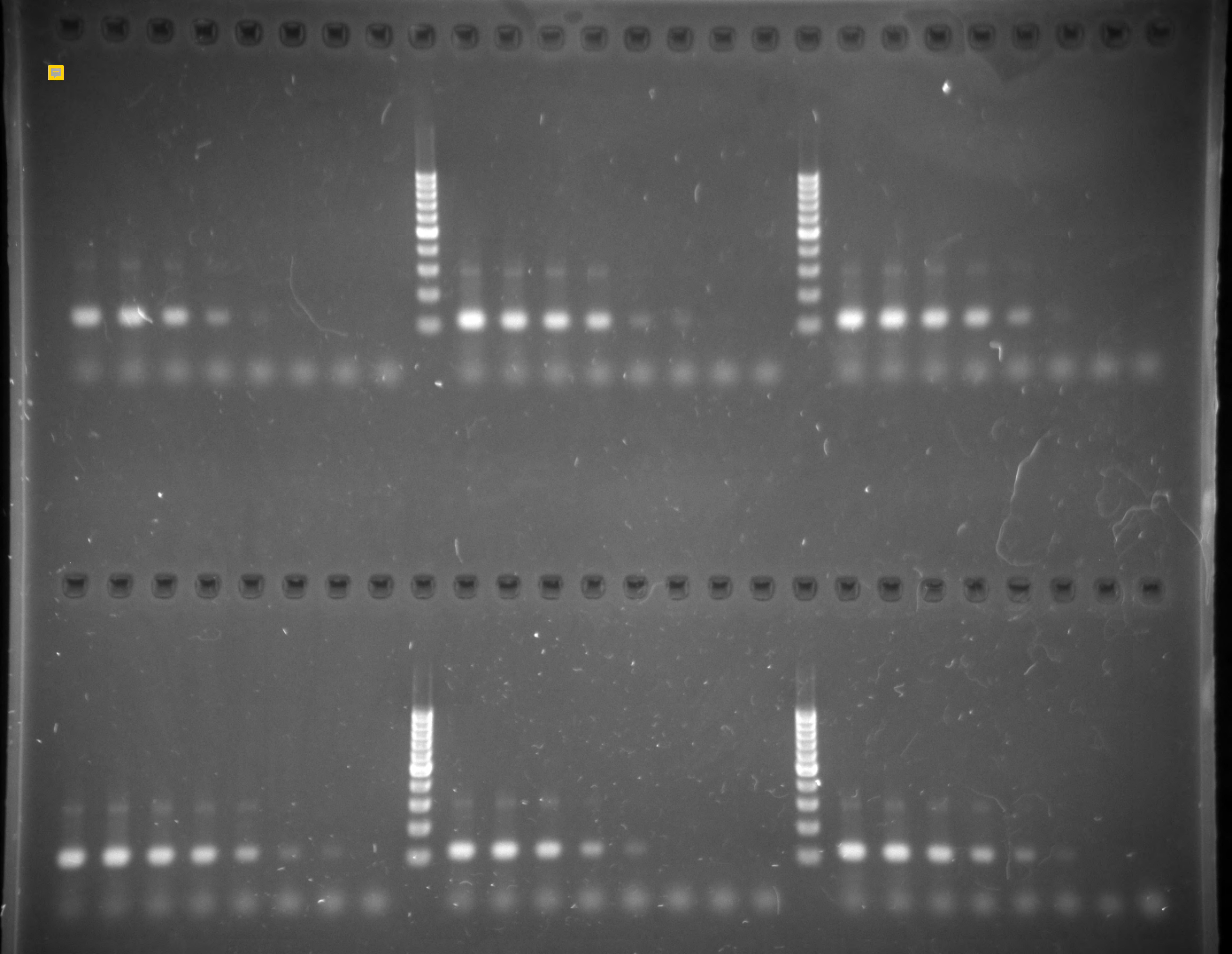

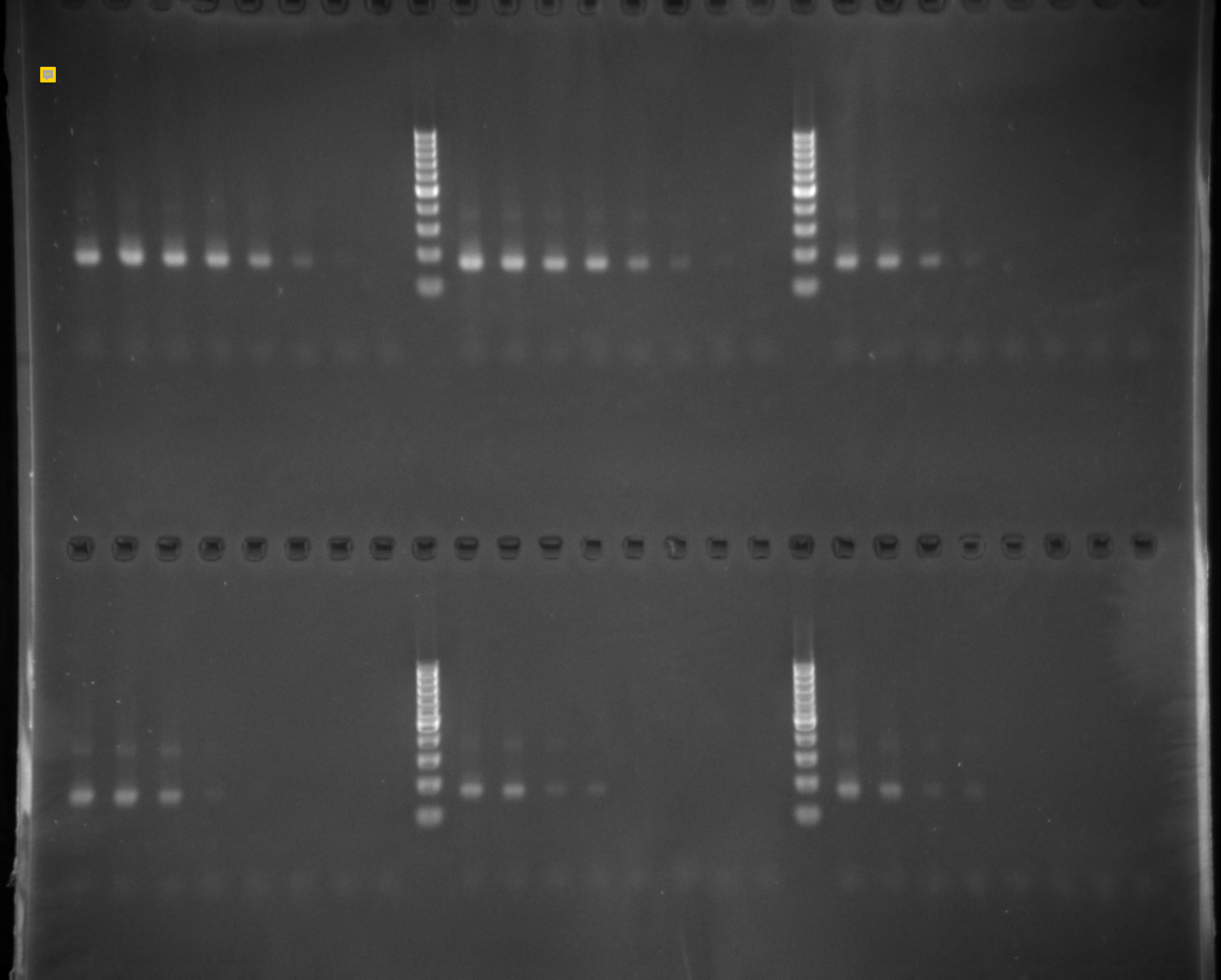

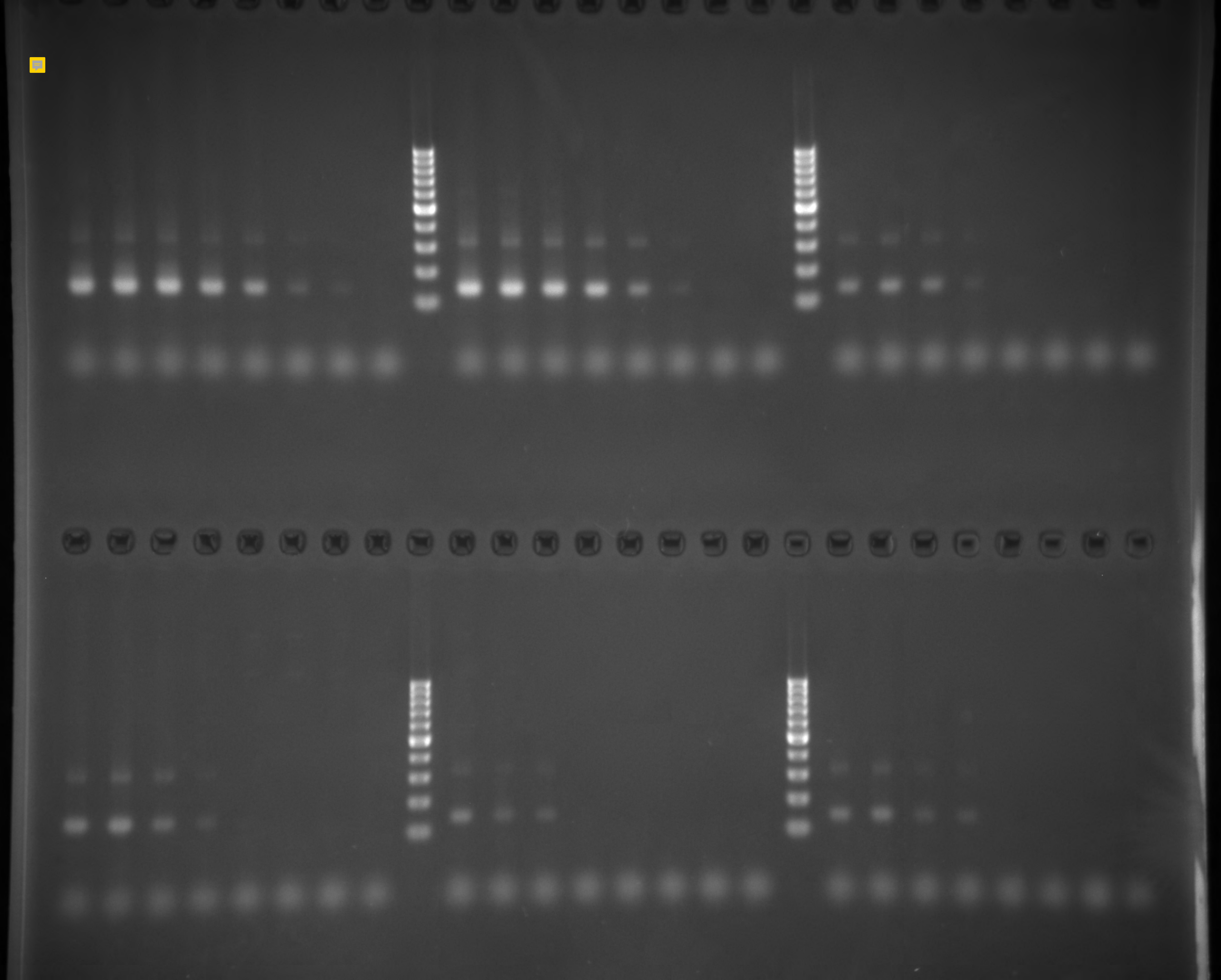

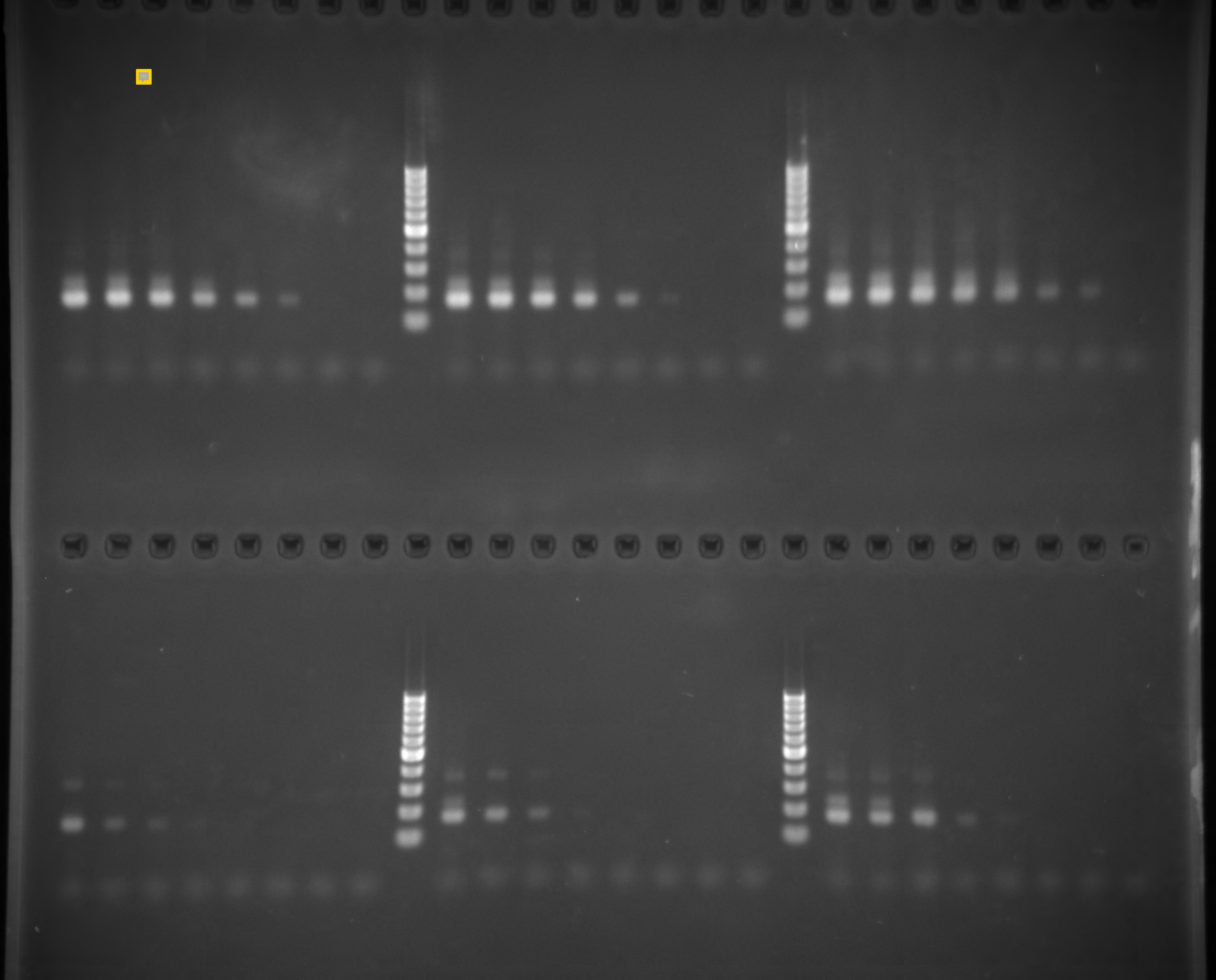

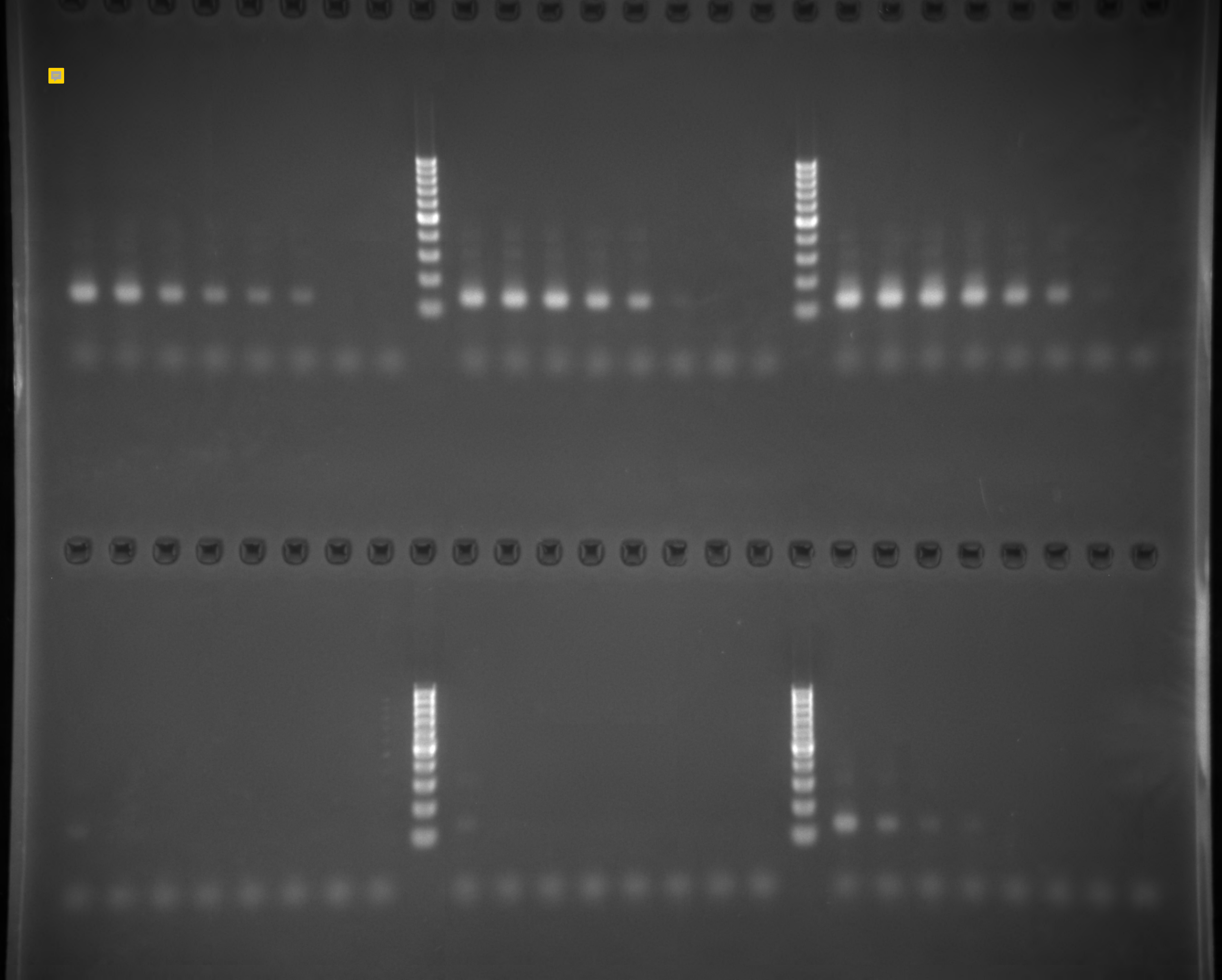

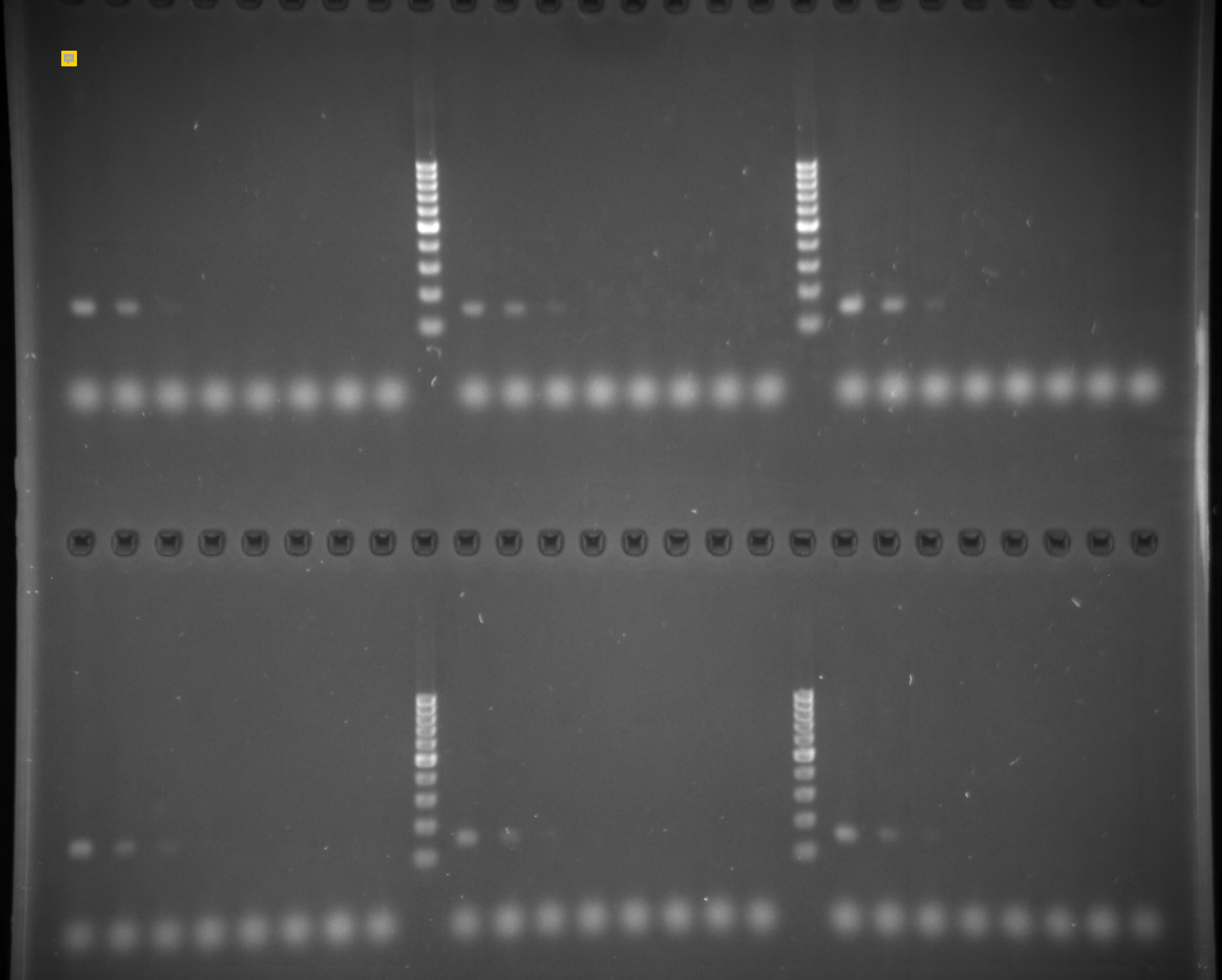

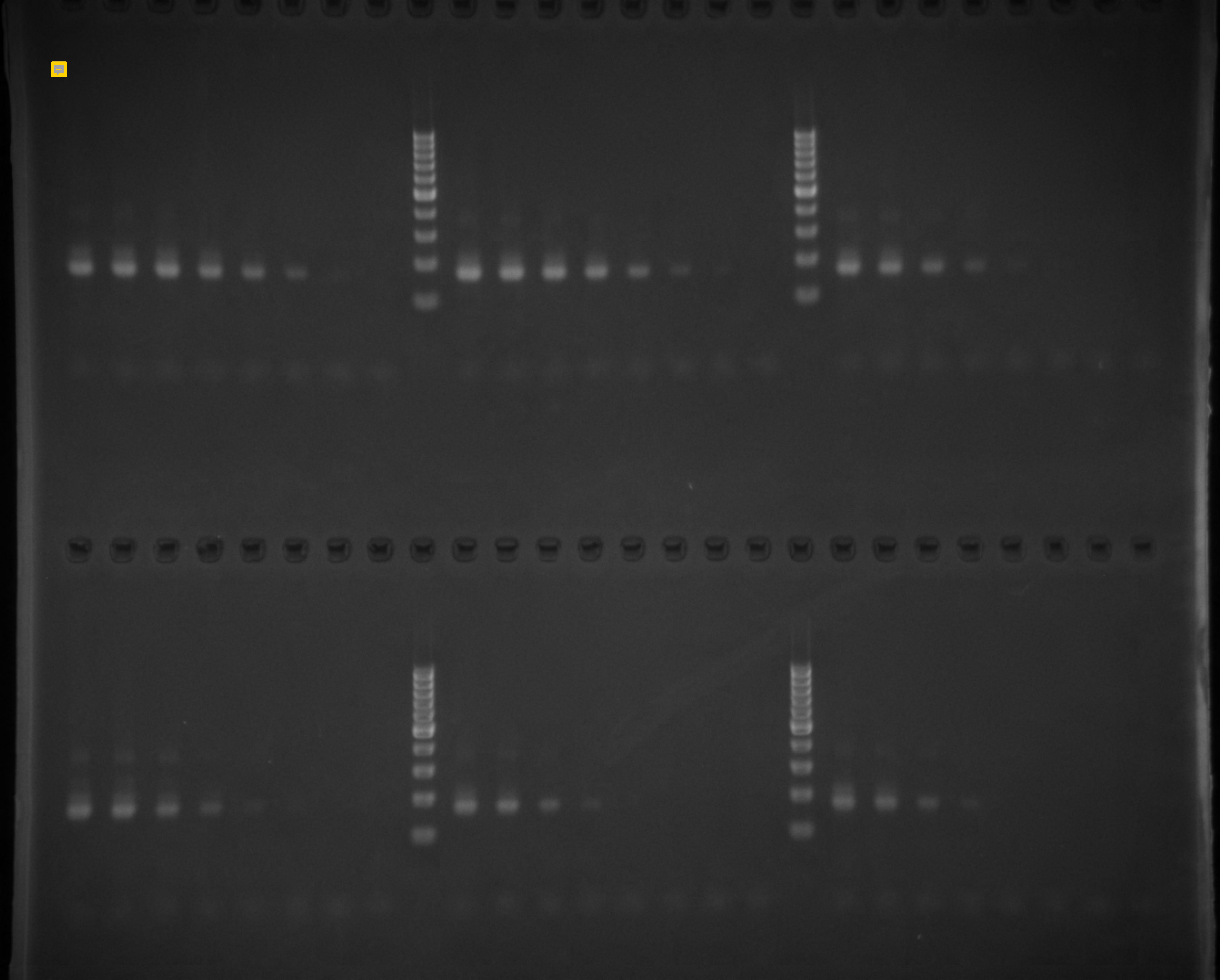

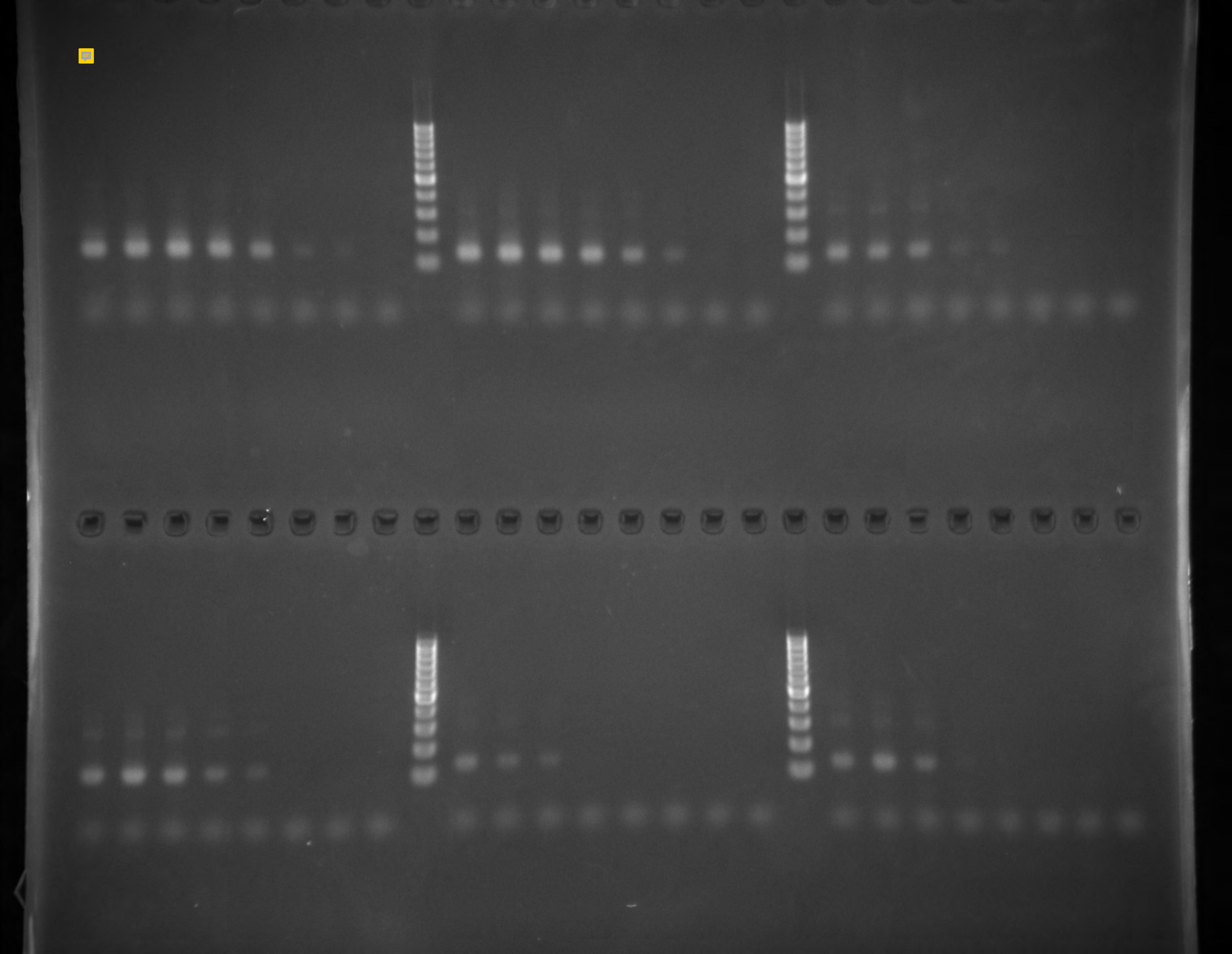

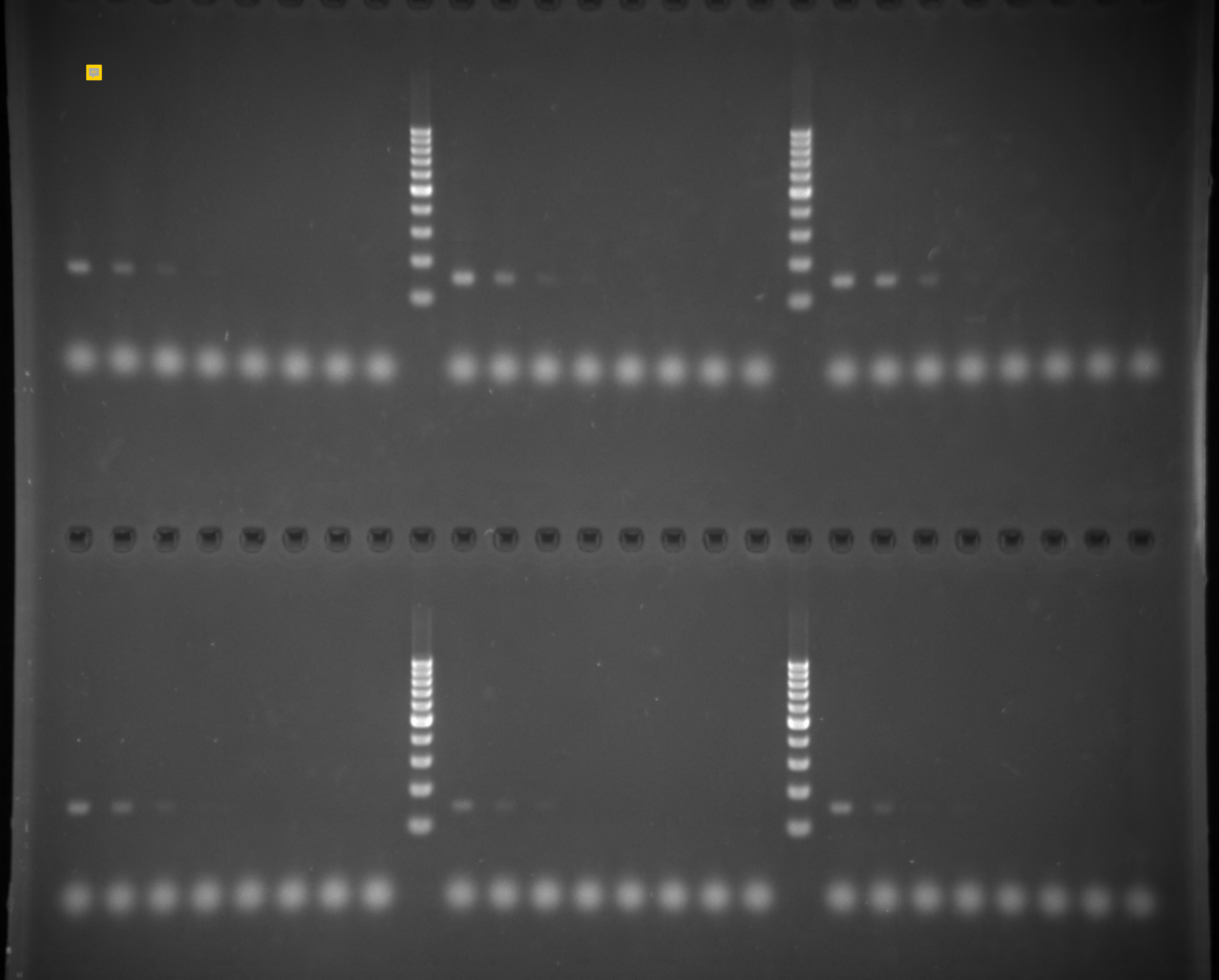

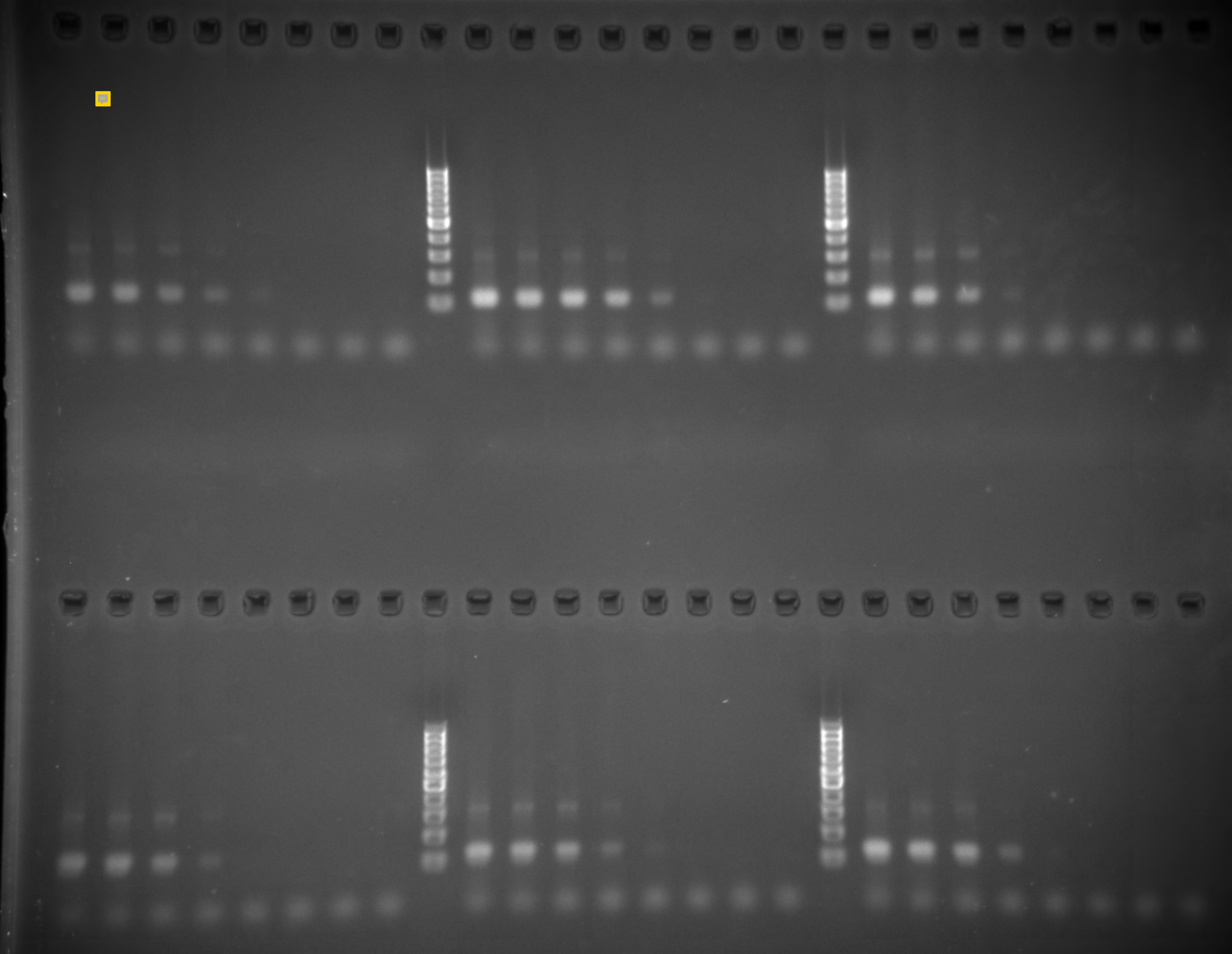

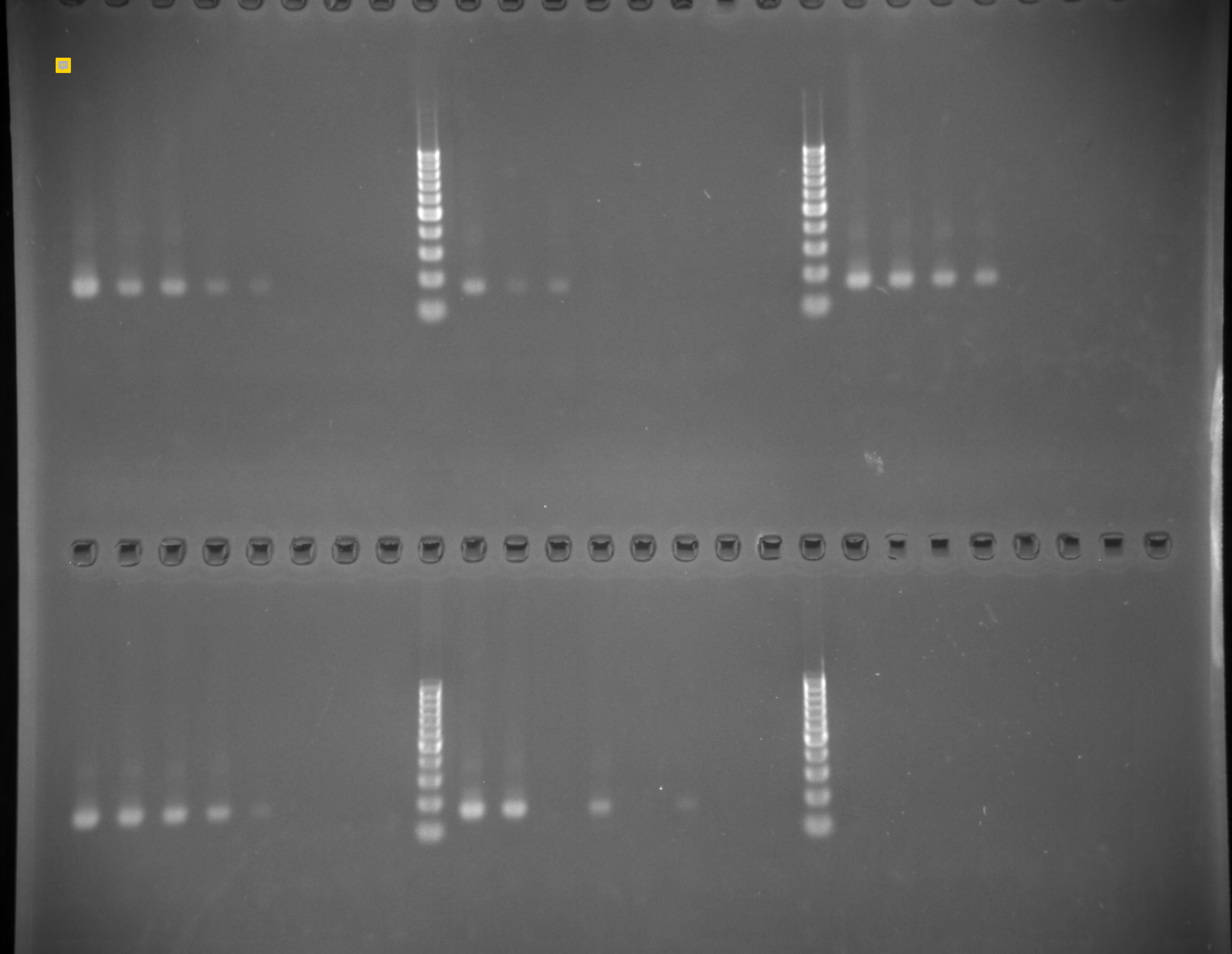

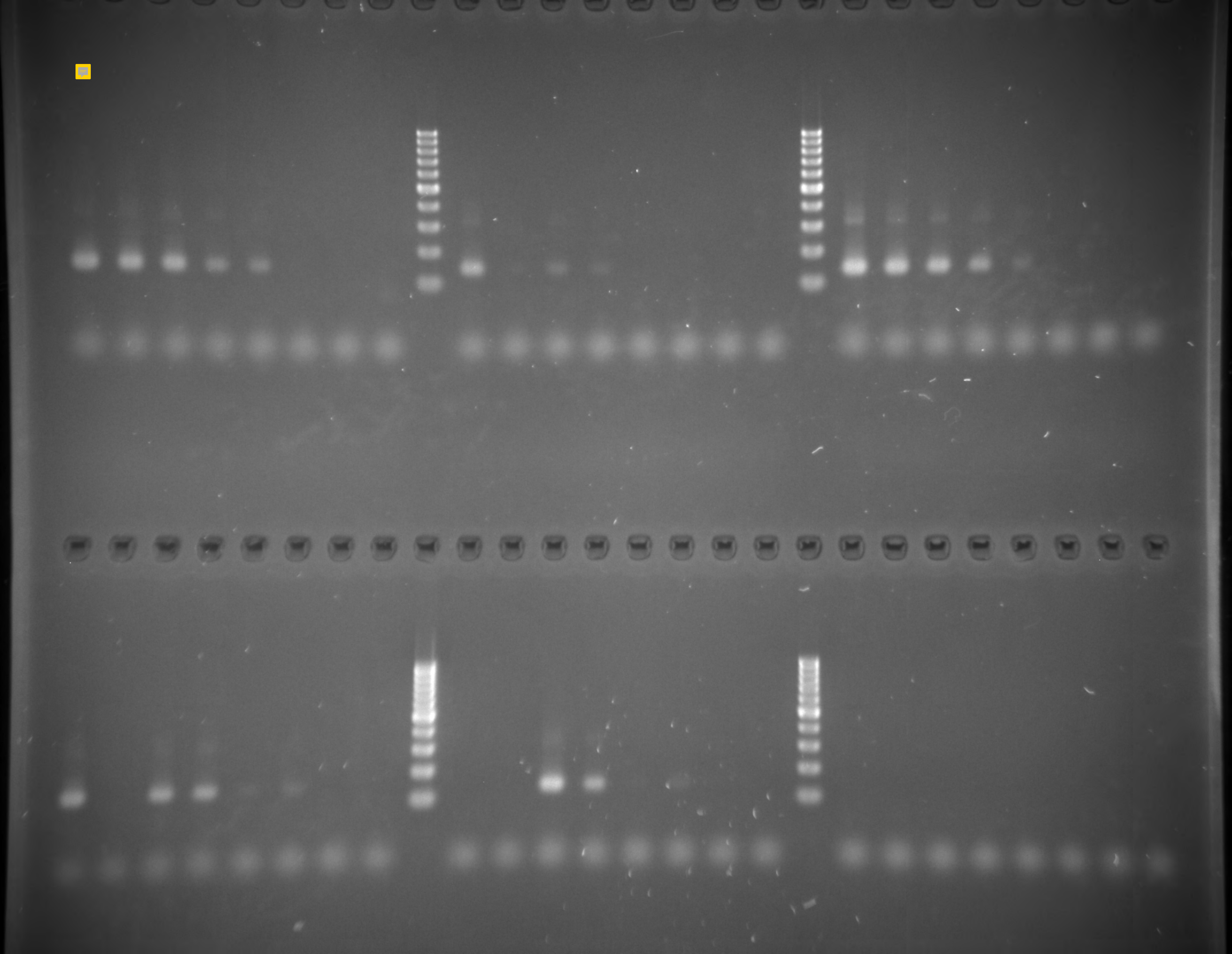

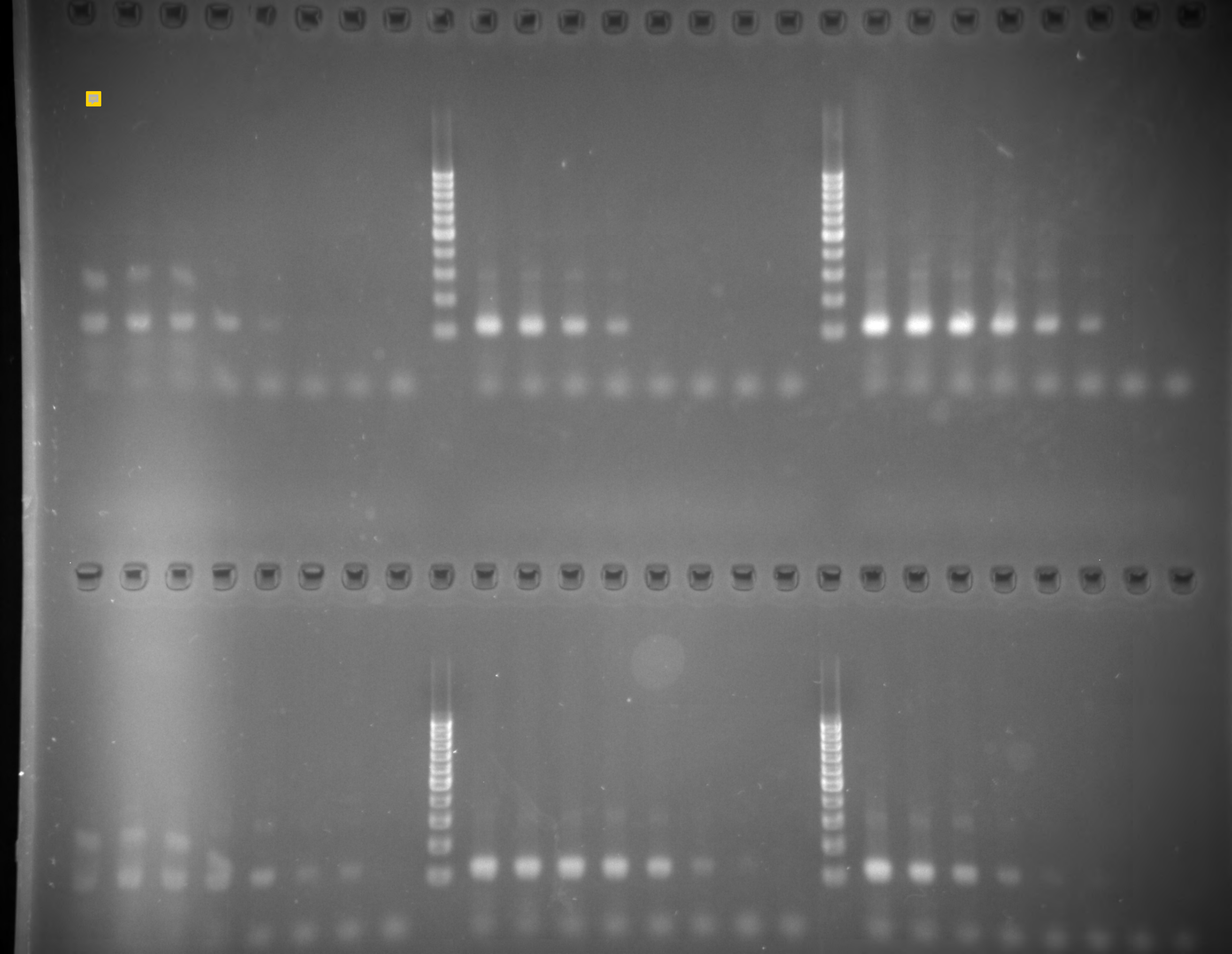

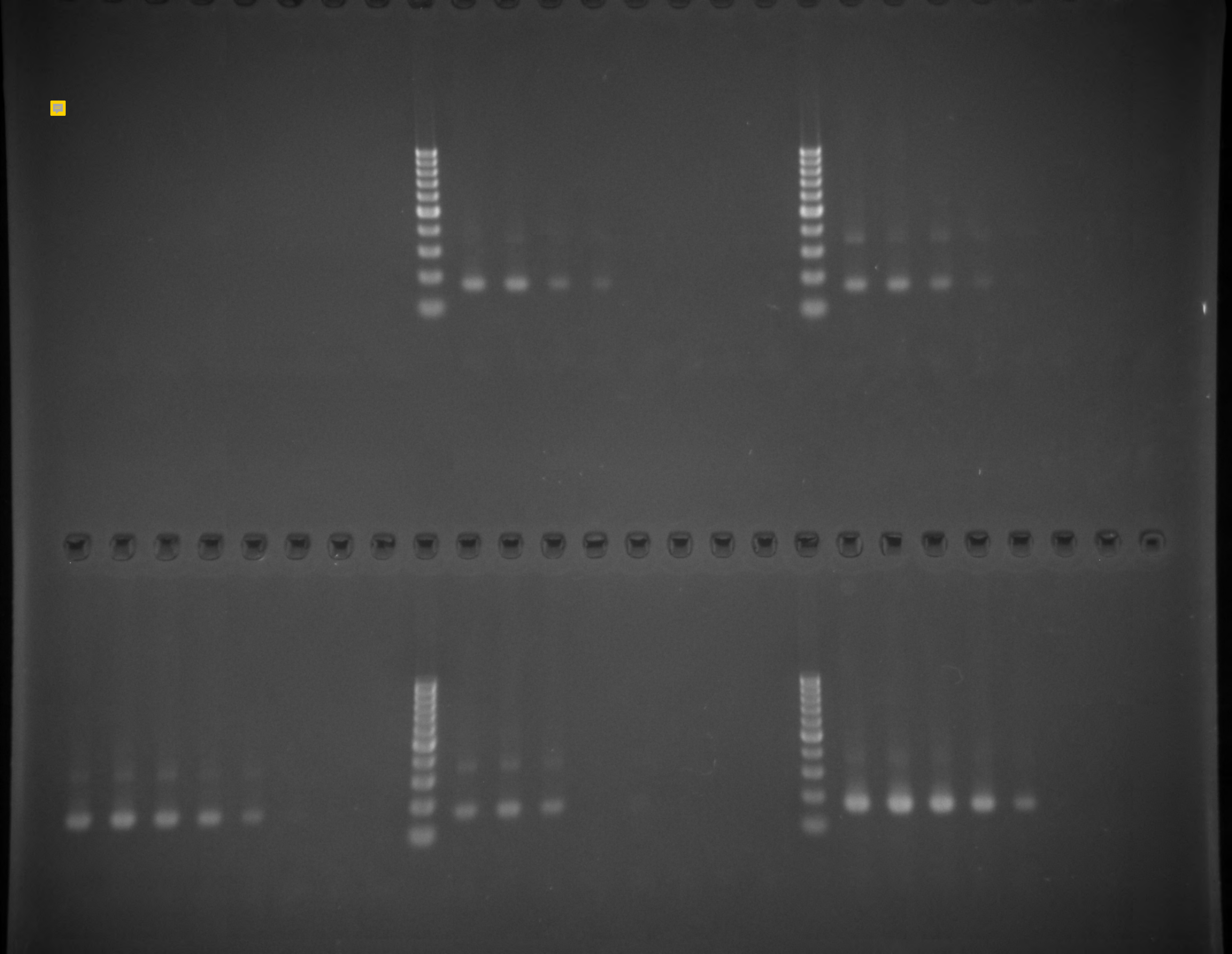

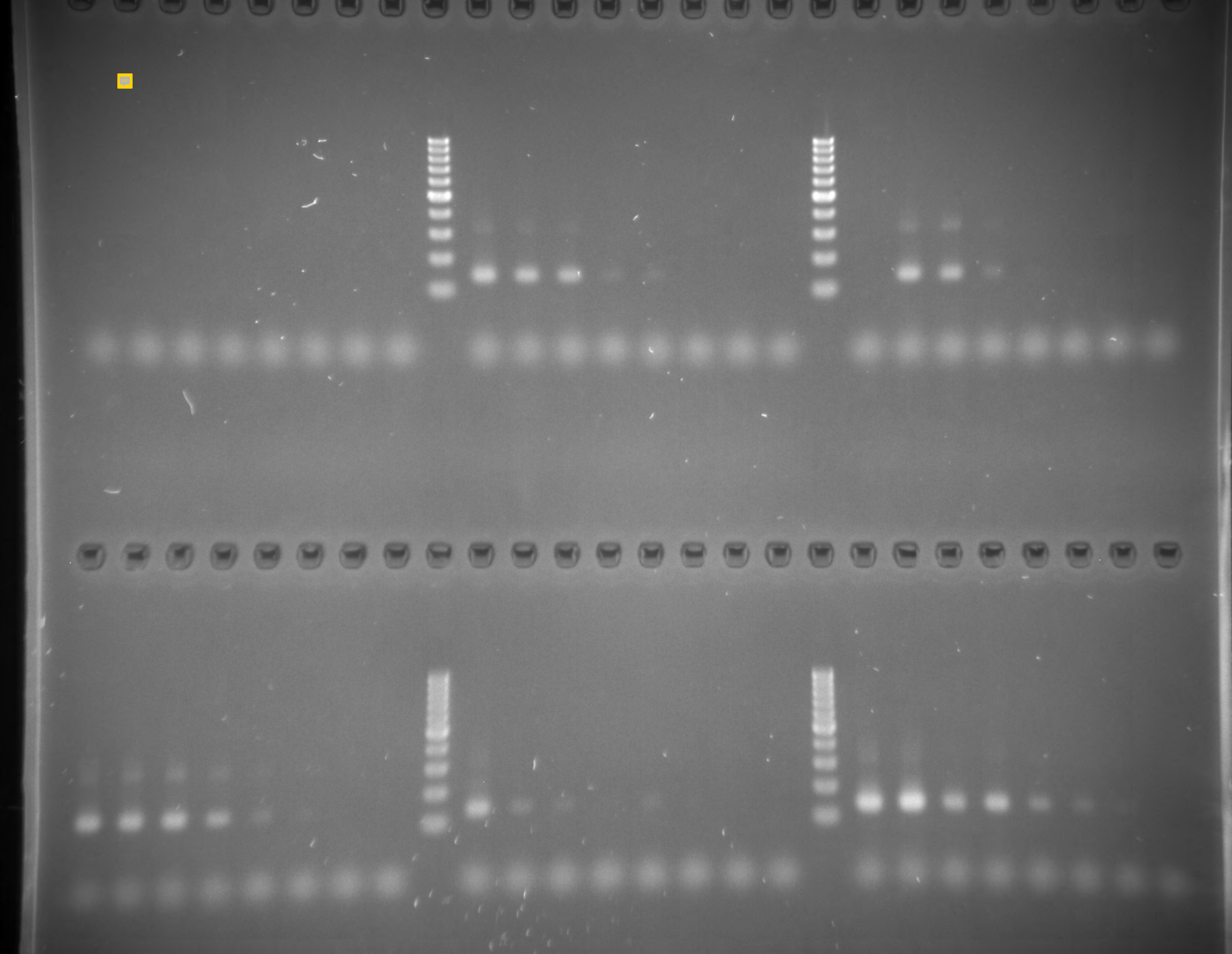

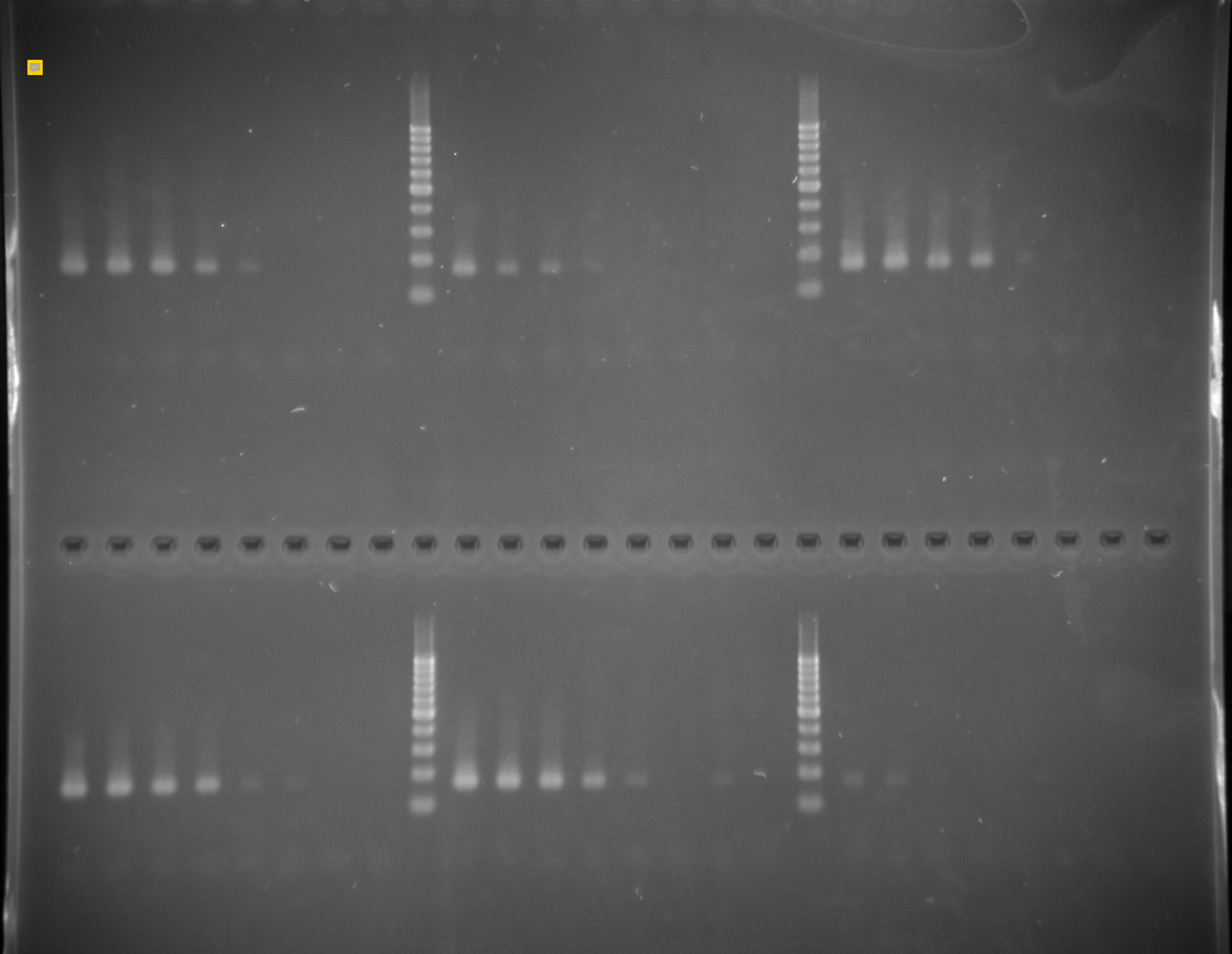

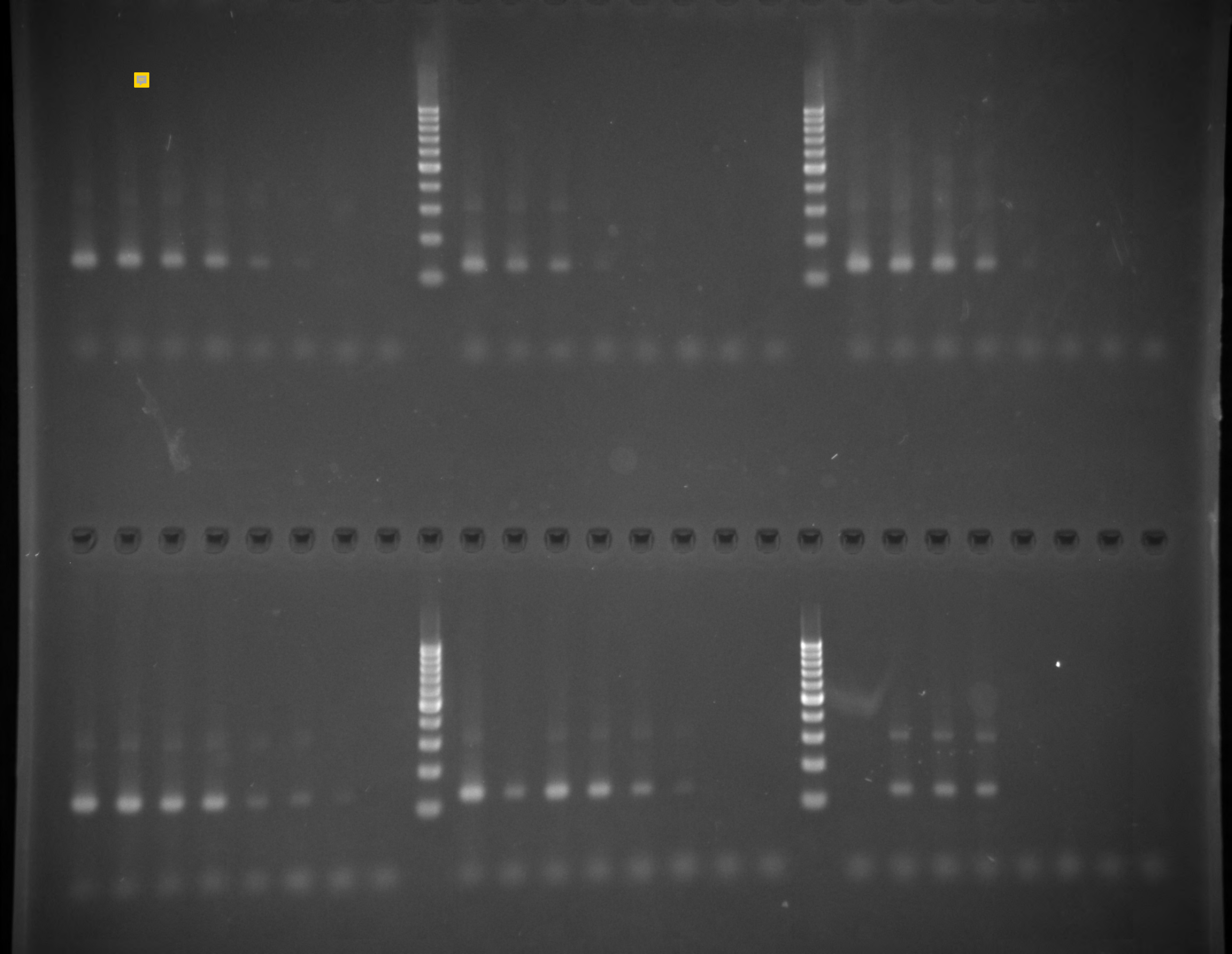

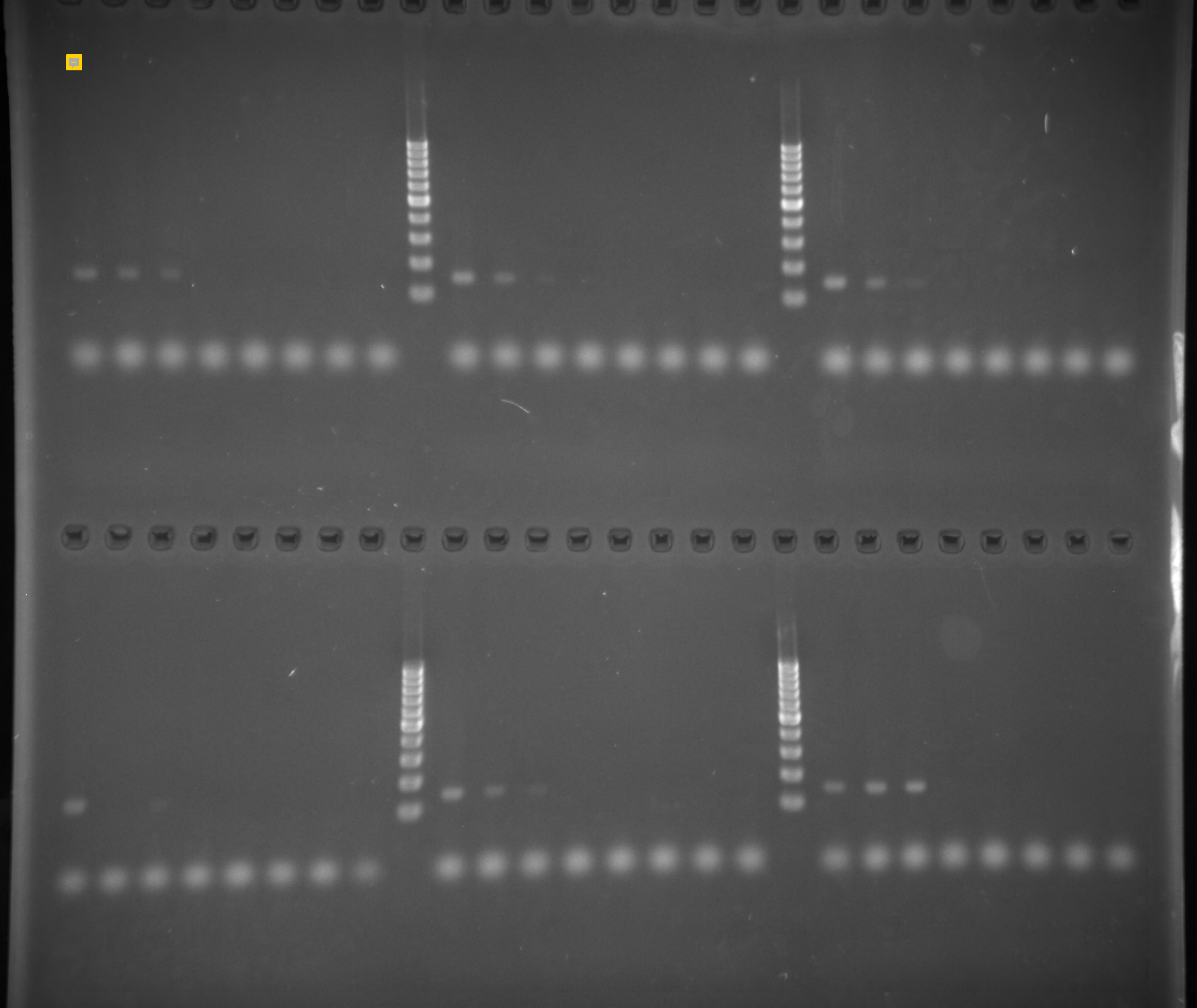

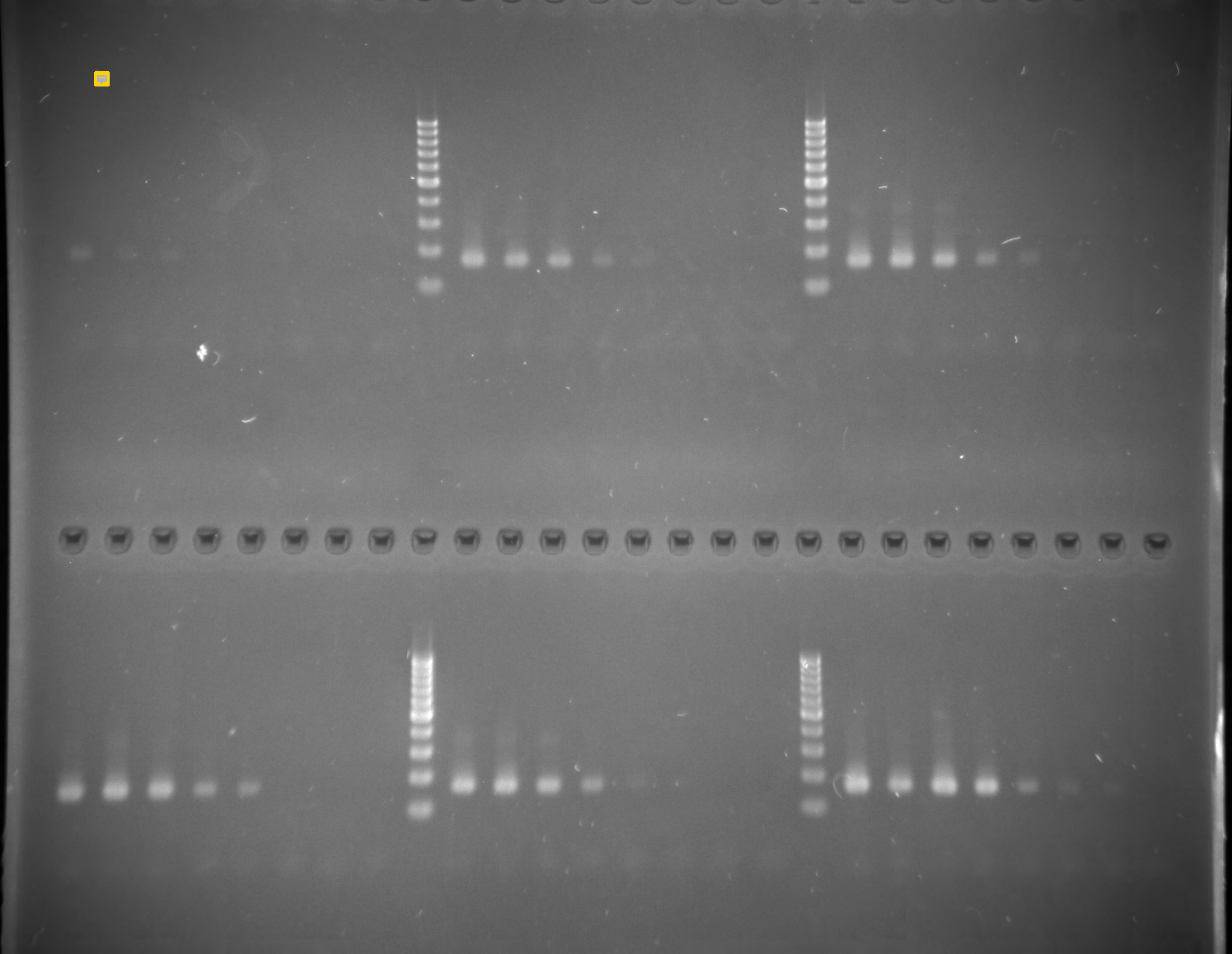

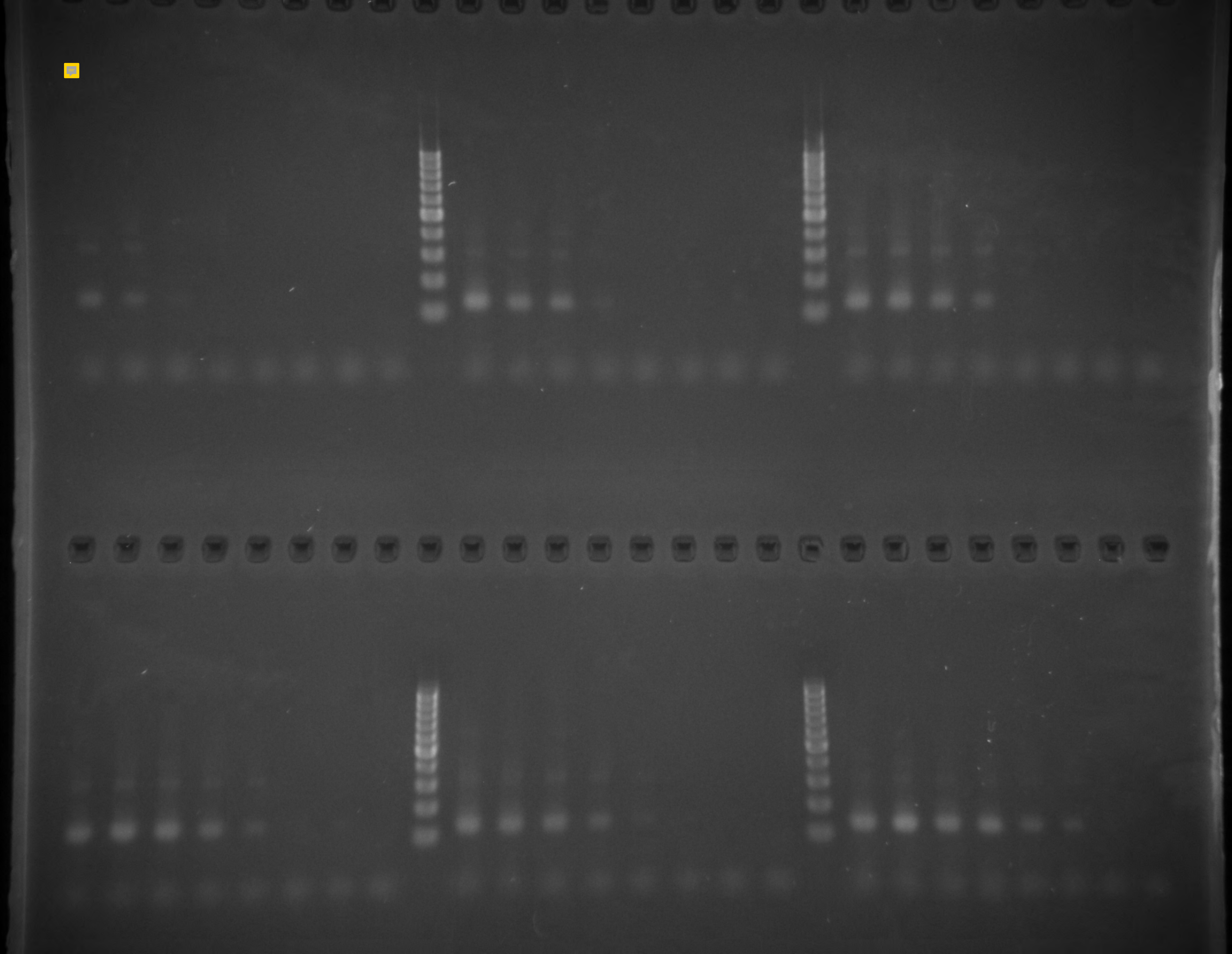

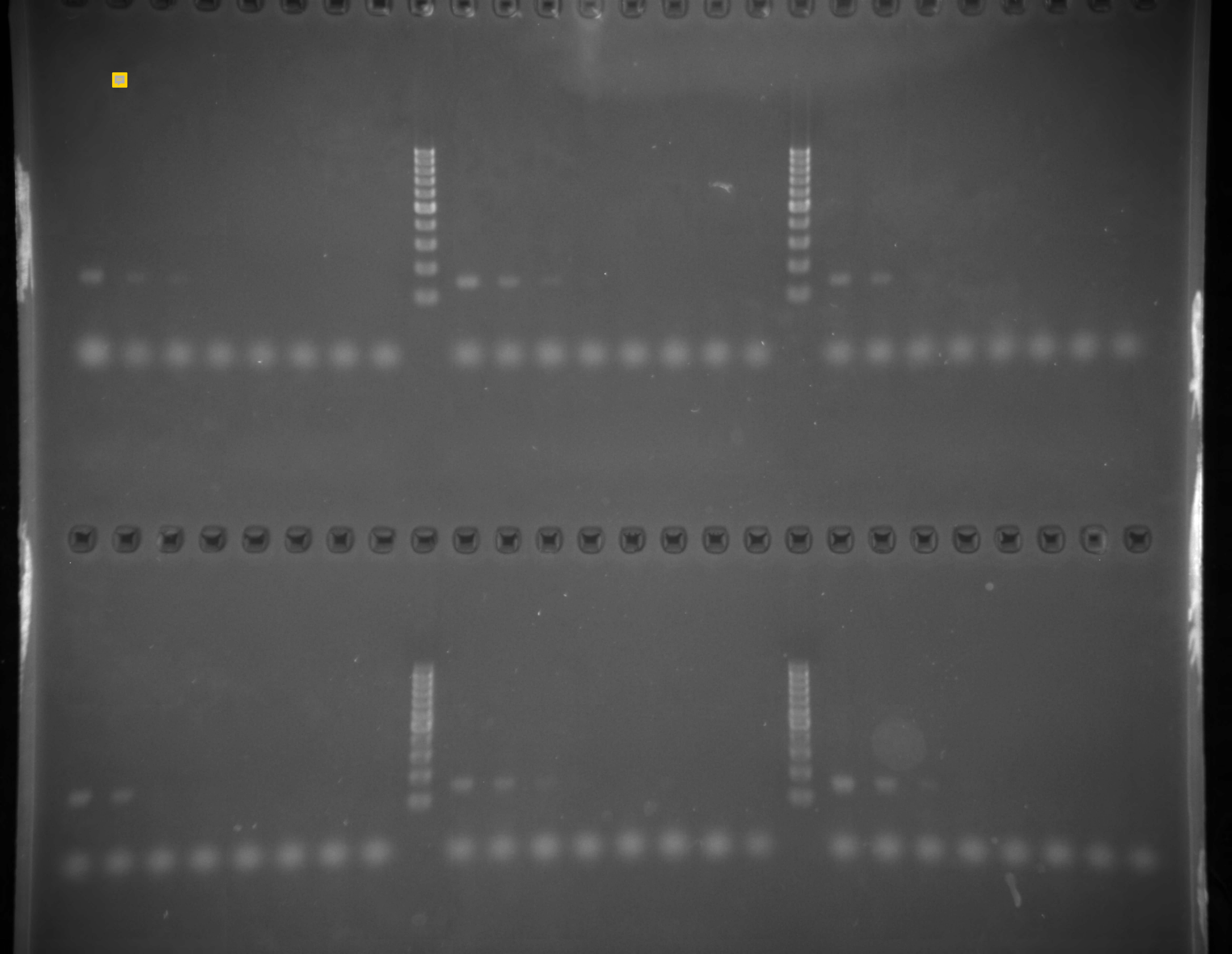

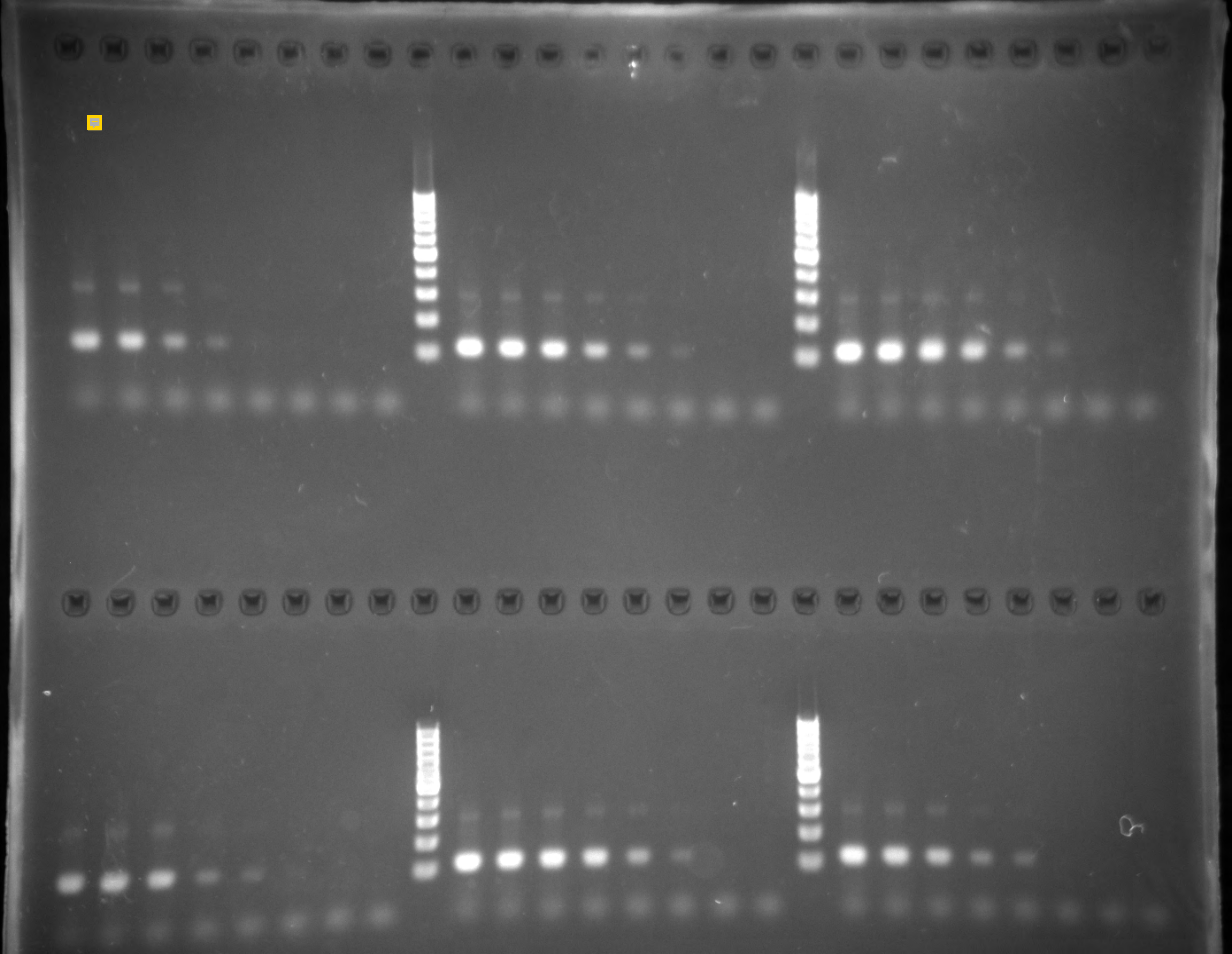

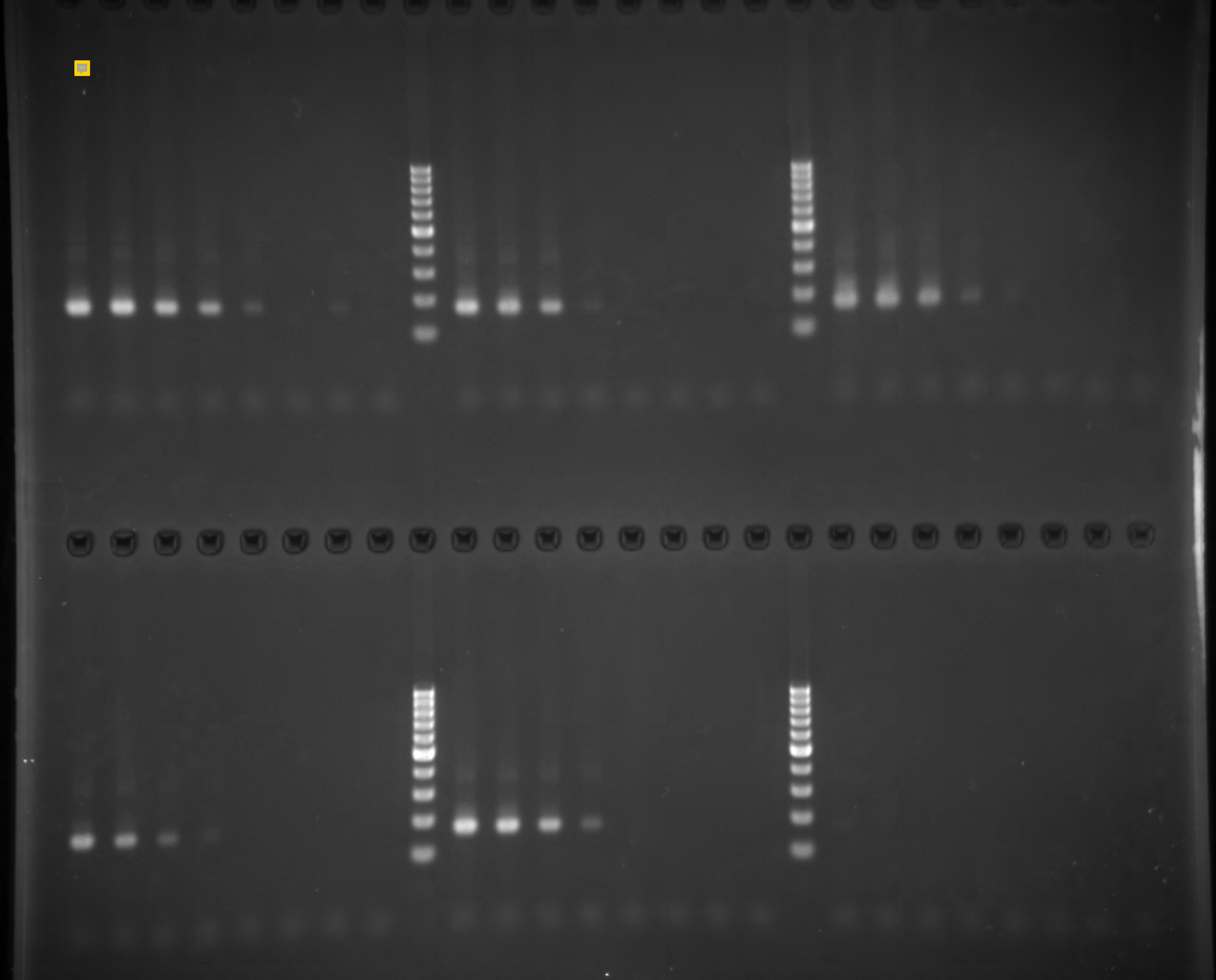

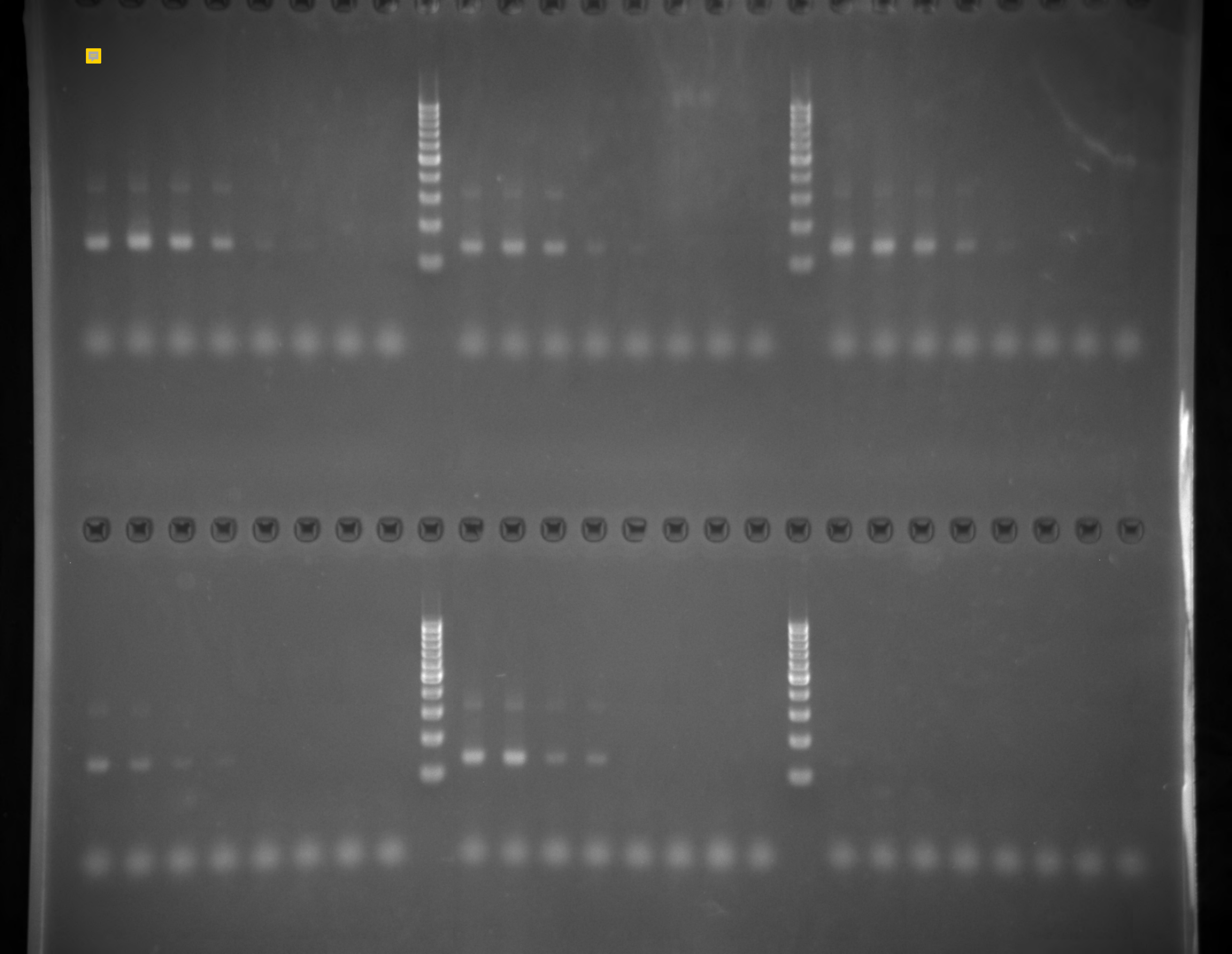

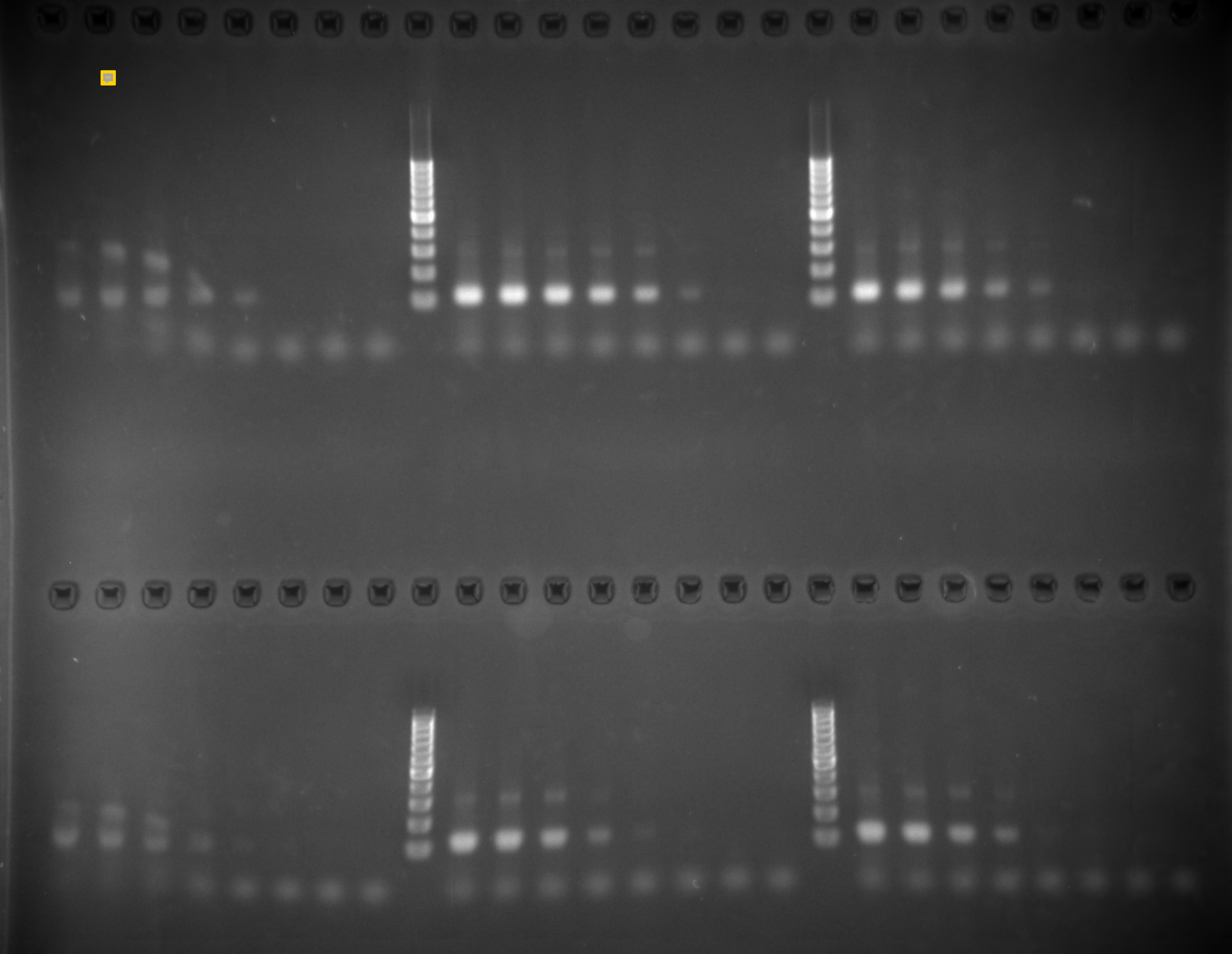

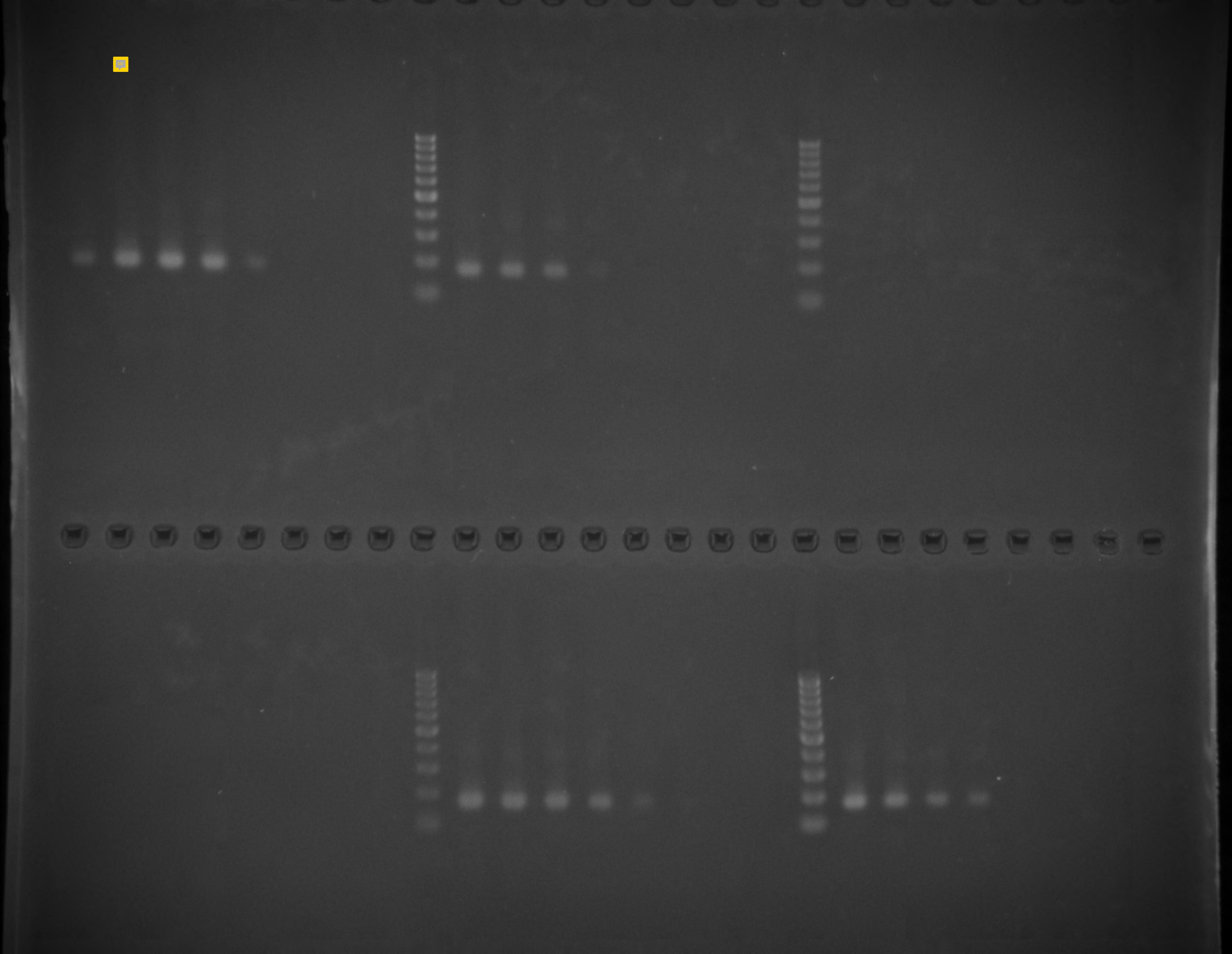

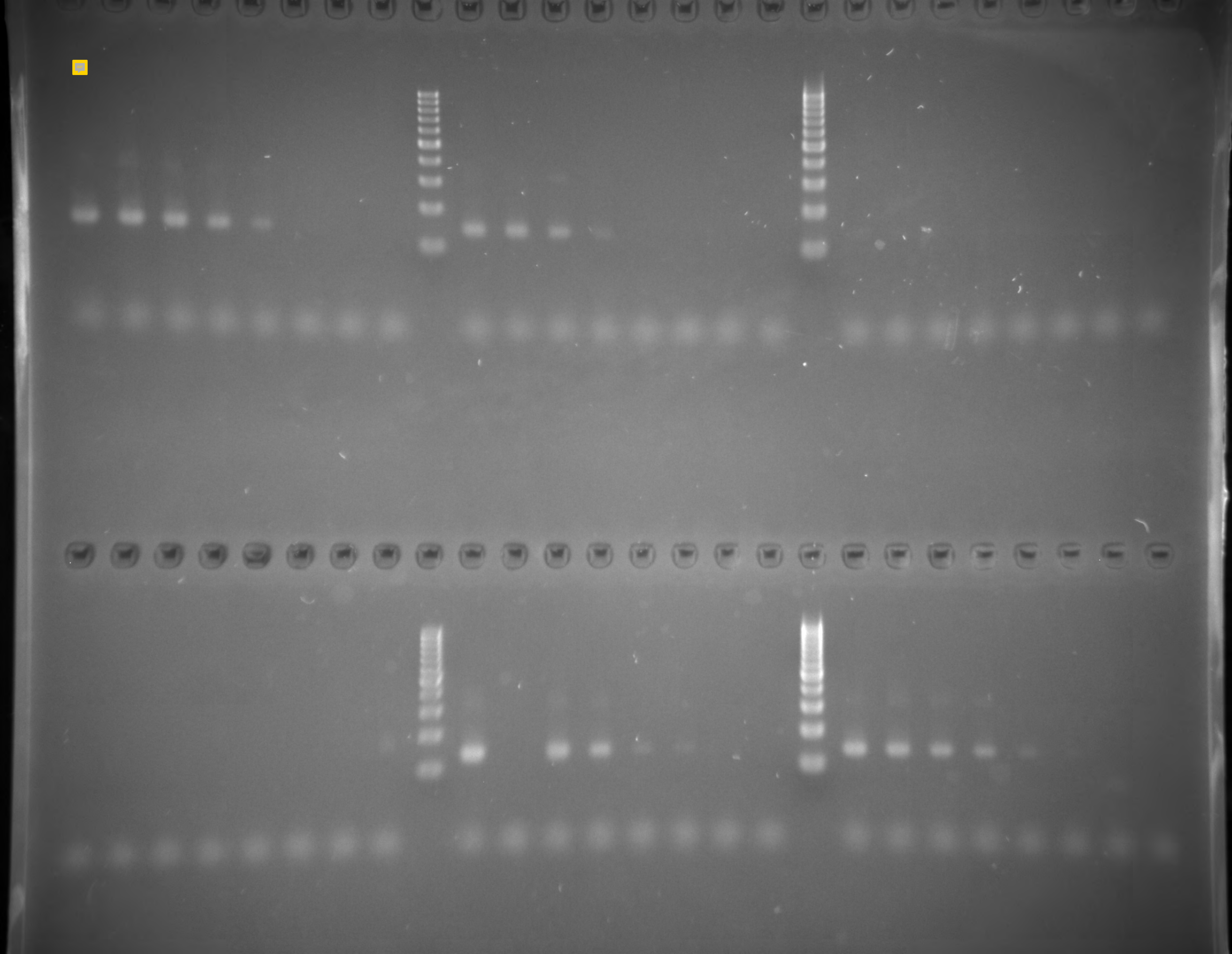

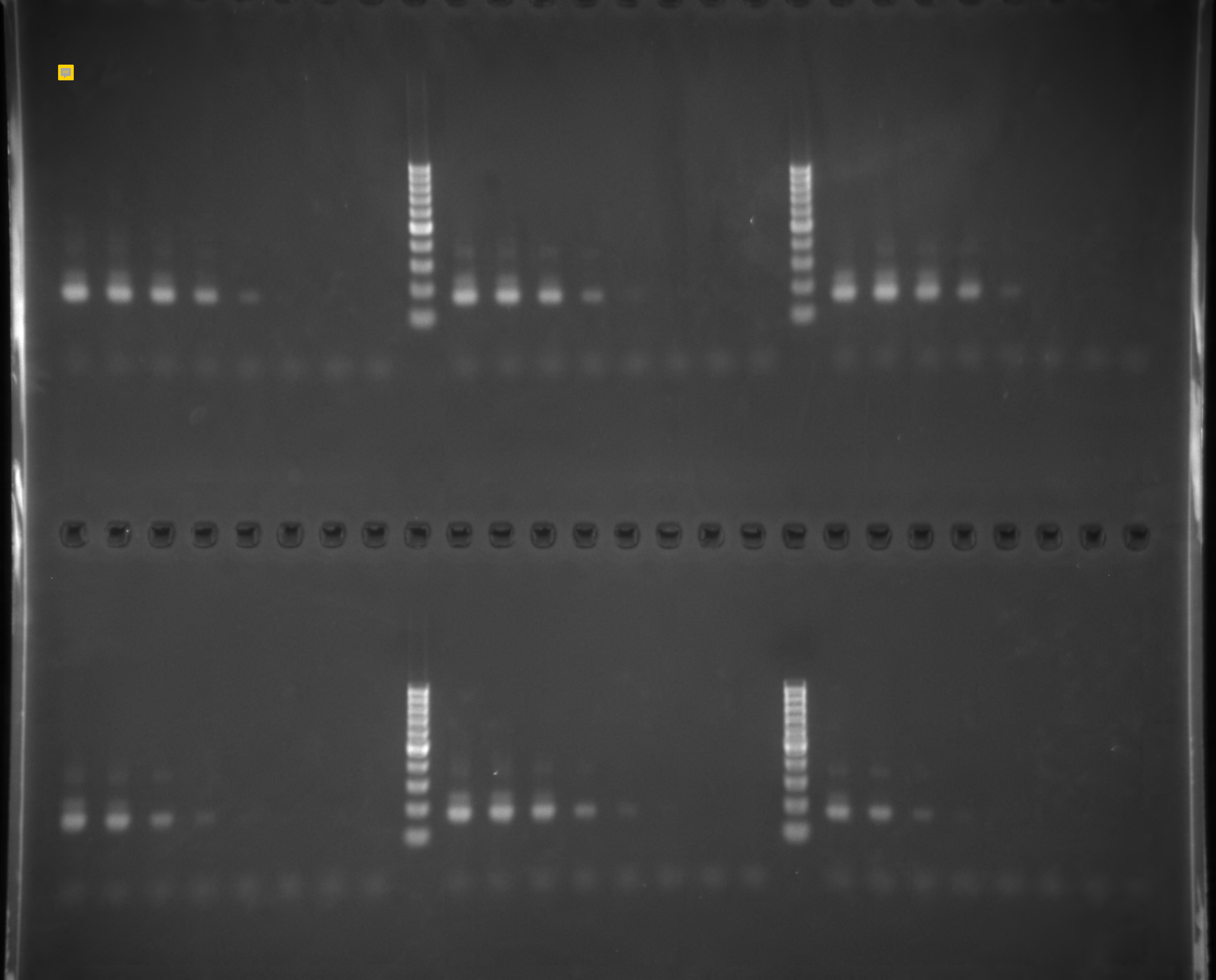

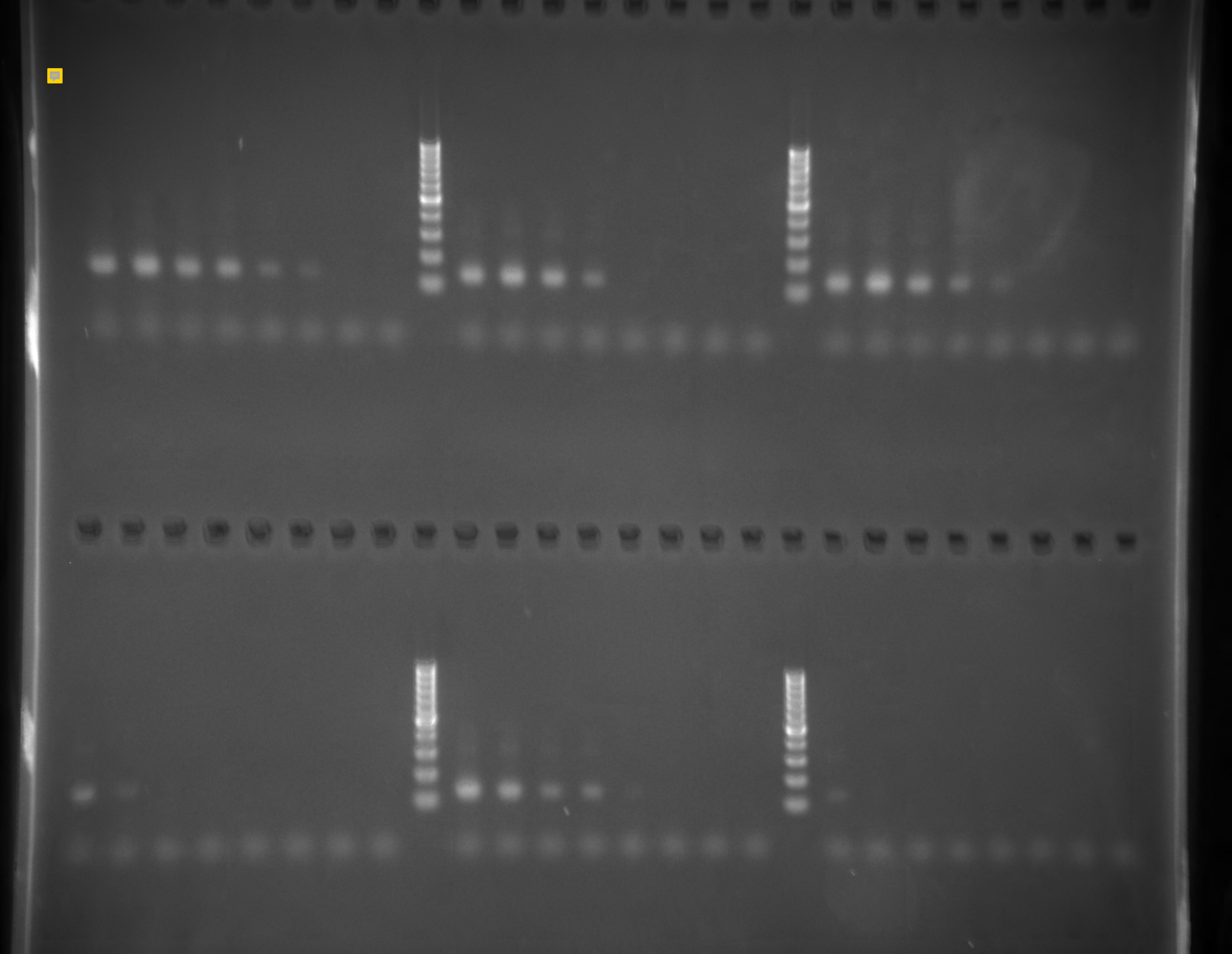

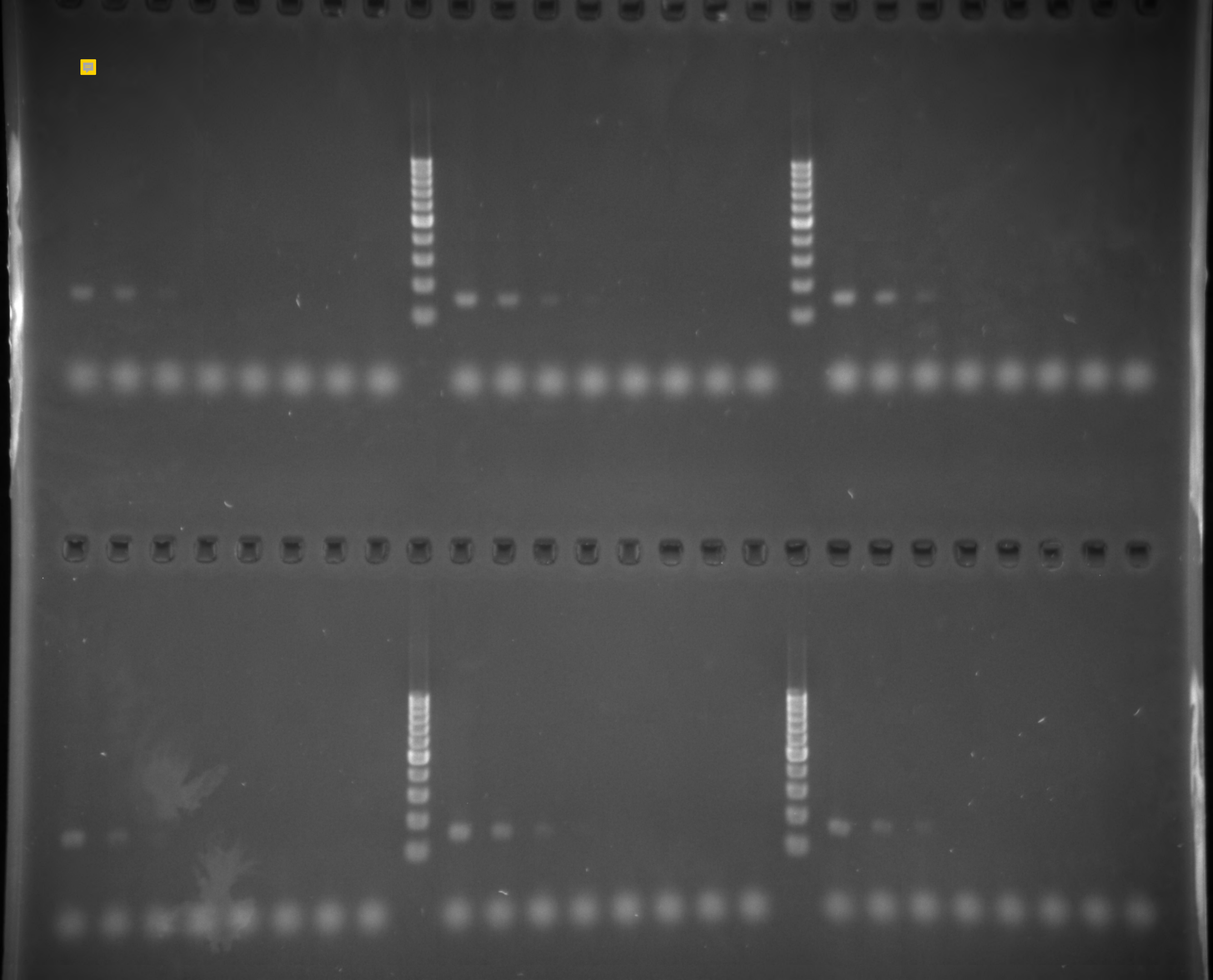

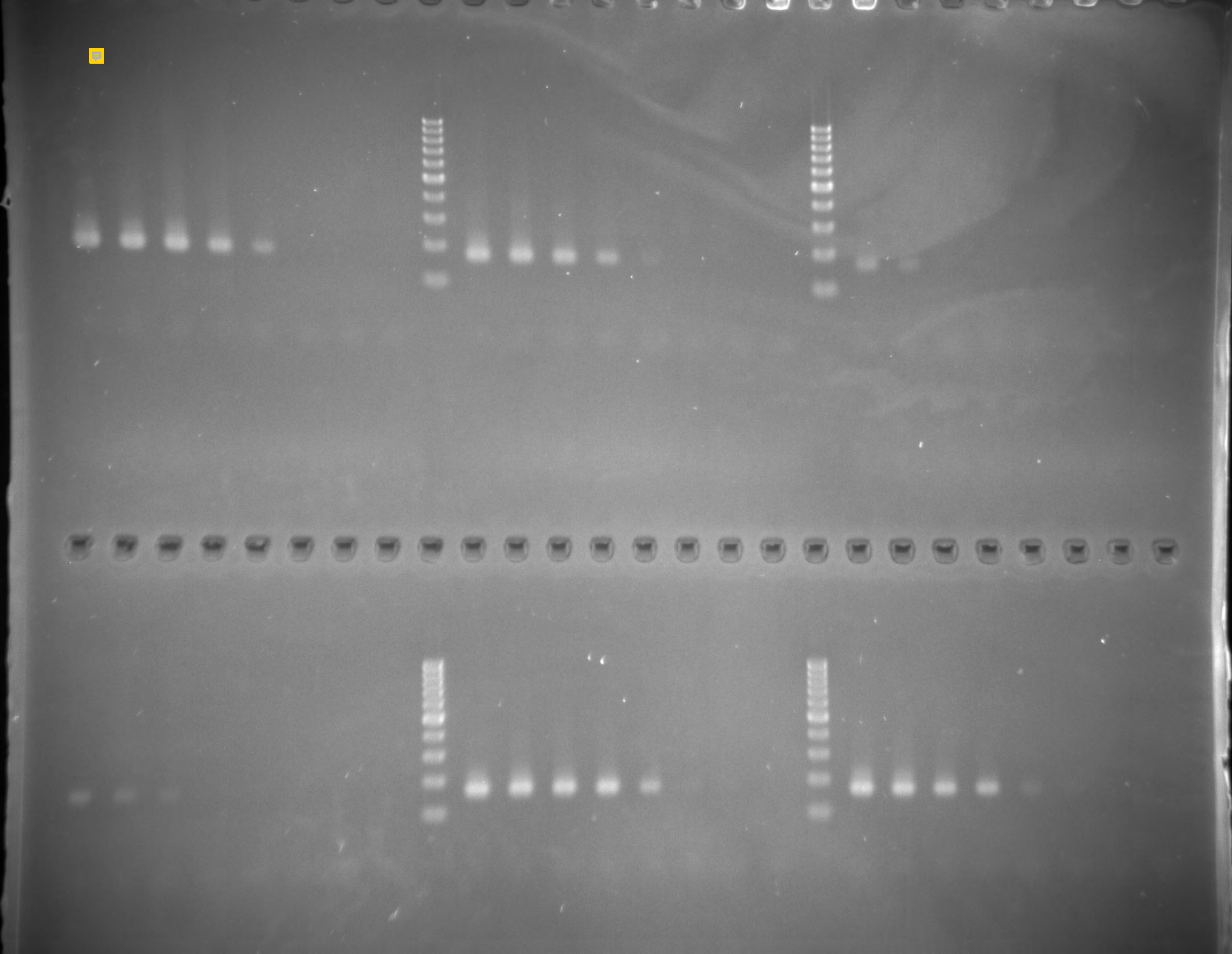

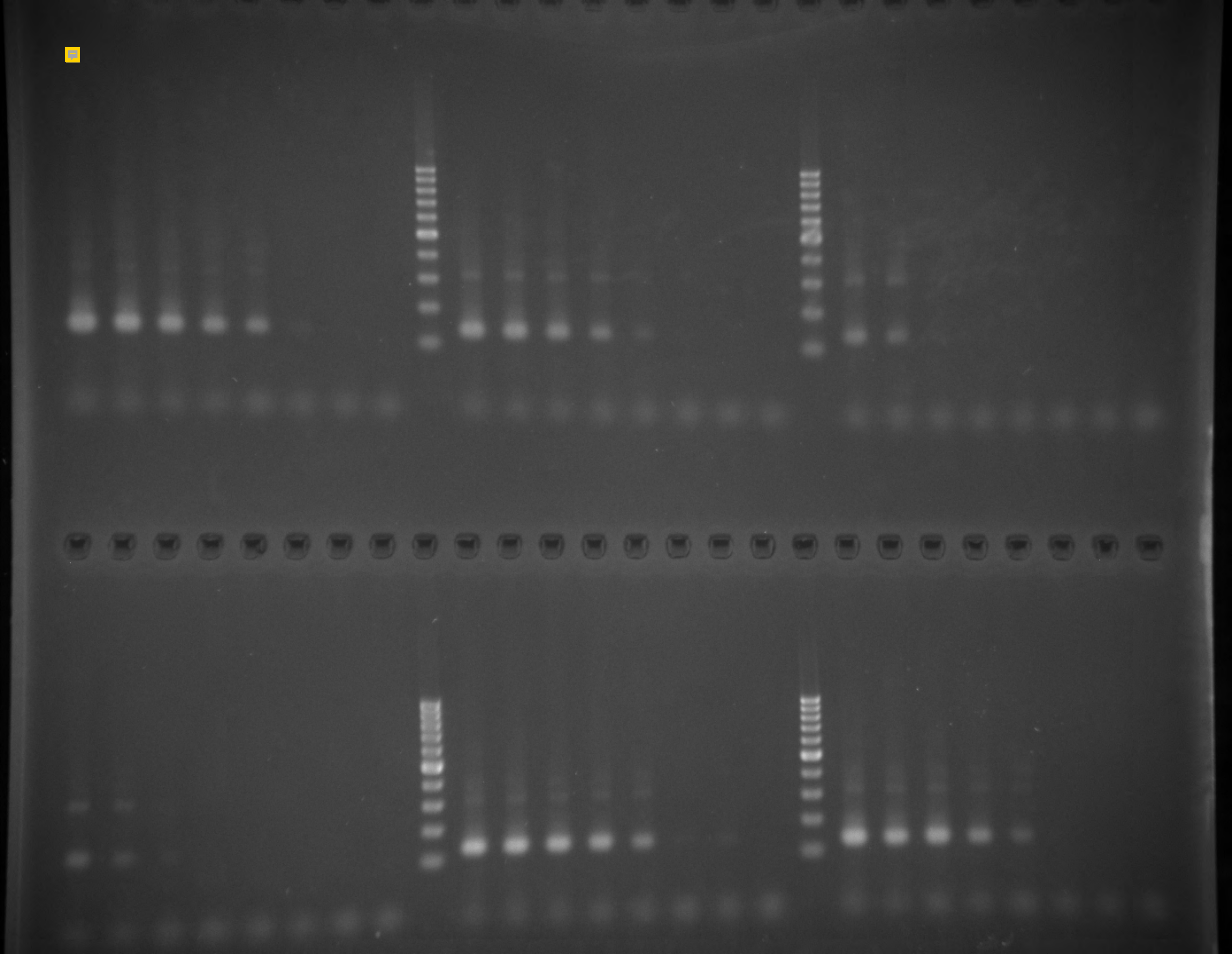

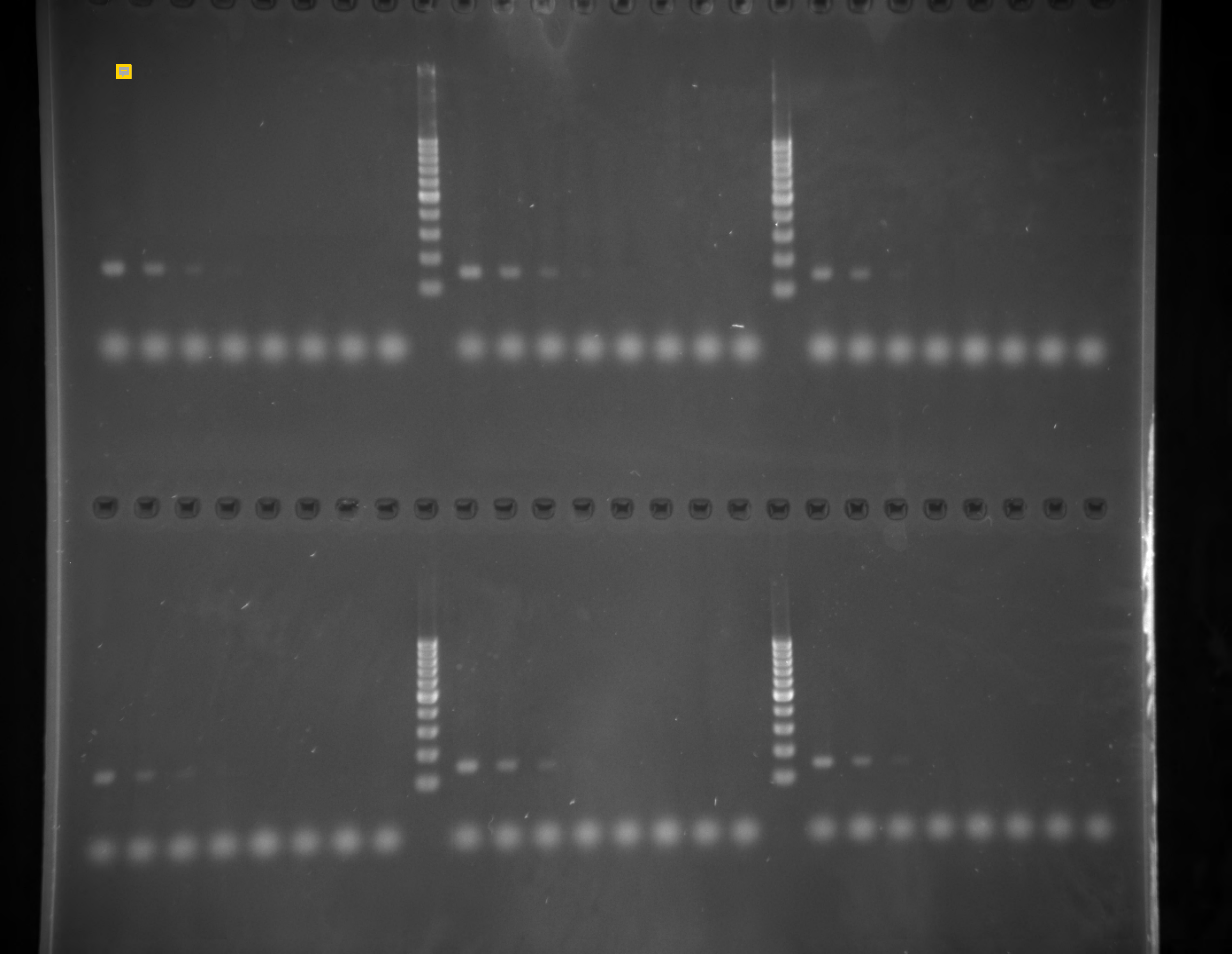

Supplement: S2 Raw images — (PDF) [file pone.0258711.s007.pdf]
