# Supplementary material for: Single nucleotide polymorphisms and copy-number variations in the Trypanosoma brucei repeat (TBR) sequence can be used to enhance amplification and genotyping of Trypanozoon strains
Source: PLoS One. 2021 Oct 25;16(10):e0258711. doi: 10.1371/journal.pone.0258711 (PMC8544829; doi:10.1371/journal.pone.0258711)
Supplement: Attachment — Submitted filename: TBR Rebuttal PONE.docx [file pone.0258711.s010.docx]

Dear Editor,

Thank you for allowing us the opportunity to present our research article to PLoS ONE, after the transfer from PLoS Neglected Tropical Diseases. Much to our surprise, our article was rejected, 4 months after we completed revision 1. We still believe that our results deserve to be published.

From the decision letter…

‘We understand you will be disappointed at this decision, which we did not reach lightly following a revision. After discussing this manuscript at some length, we considered that further revisions are unlikely to address the major concern that the presented results give little insight into the genome-wide diversity or evolution of the repeats, as this is difficult to gather from the methodological approach. Given that the presented method also seems technically difficult for a surveillance tool in a close-to-the-clinic setting, both the editors and reviewers consider that this manuscript is unlikely to be of sufficiently wide interest to the PLoS NTD readership.’

The Editor of PLoS NTD is correct to state that our methodological current is not able to give further insight into the genome-wide diversity or evolution of the repeats. However, this was not the aim of the manuscript. Our point is that the TBR sequence is not a homogenous sequence. Our most important finding is that our novel PCR allows a more sensitive detection of *T.b. gambiense* and *T. evansi* compared to other TBR PCRs due to the fact that the TBR content of these taxa is different.

We do not understand which requirements the Editor requests ‘for a surveillance tool in a close-to-the-clinic setting’. However, considering that we present novel primers and probes, the current proposed qPCR format, which is indeed performed in a laboratory setting, may be transferred to a more innovative molecular diagnostic tool that can be performed at the point-of-care. Again, this was not the aim of the article.

Reviewer's Responses to Questions

<b>Key Review Criteria Required for Acceptance?</b></br></br> As you describe the new analyses required for acceptance, please consider the following:</br></br>

<b>Methods</b></br></br>

-Are the objectives of the study clearly articulated with a clear testable hypothesis stated?</br> -Is the study design appropriate to address the stated objectives?</br> -Is the population clearly described and appropriate for the hypothesis being tested?</br> -Is the sample size sufficient to ensure adequate power to address the hypothesis being tested?</br> -Were correct statistical analysis used to support conclusions?</br> -Are there concerns about ethical or regulatory requirements being met?</br></br>

Reviewer #1: The methods are adequately described.

Reviewer #3: (No Response)

--------------------

<b>Results</b></br></br>

-Does the analysis presented match the analysis plan?</br> -Are the results clearly and completely presented?</br> -Are the figures (Tables, Images) of sufficient quality for clarity?</br></br>

Reviewer #1: The results clearly are and completely presented

Reviewer #3: (No Response)

--------------------

<b>Conclusions</b></br></br>

-Are the conclusions supported by the data presented?</br> -Are the limitations of analysis clearly described?</br> -Do the authors discuss how these data can be helpful to advance our understanding of the topic under study?</br> -Is public health relevance addressed?</br></br>

Reviewer #1: The conclusions are supported by the data presented. The public health relevance is addressed.

Reviewer #3: (No Response)

--------------------

<b>Editorial and Data Presentation Modifications?</b></br><br/> Use this section for editorial suggestions as well as relatively minor modifications of existing data that would enhance clarity. If the only modifications needed are minor and/or editorial, you may wish to recommend “Minor Revision” or “Accept”.

Reviewer #1: (No Response)

Reviewer #3: (No Response)

--------------------

<b>Summary and General Comments</b></br></br> Use this section to provide overall comments, discuss strengths/weaknesses of the study, novelty, significance, general execution and scholarship. You may also include additional comments for the author, including concerns about dual publication, research ethics, or publication ethics. If requesting major revision, please articulate the new experiments that are needed.

Reviewer #1: My main concern remains the same. I think the presented manuscript is of limited interest to the audience of PLoS Neglected Tropical Diseases.

Reviewer #3: the manuscript has not substantially changed. It is not reduced in size and there is no insight into evolution of the 177bp repeat. I could not find the rebuttal letter either giving a point by point response.

--------------------

In response to the Editor and Reviewers, we made our article considerable shorter and hopefully more concise and interesting, also for a more general public. In essence, it is a story about a historical sequence that has still some interesting points to reveal. We believe that our novel PCR sets will absolutely make a difference in molecular diagnosis of T.b. gambiense and T. evansi and have good faith that these novel primers and probes will be applied on the field.

In rephrasing our article for PLoS One, we cleaned-up huge areas of text and rephrased most sentences for better clarity. I addition, we shortened the manuscript by removing unsuccessful attempts that tried to further define the observed TBR genotypes in view of surface antigens and drug resistance genes. For this reason, the ‘Revised Manuscript with Tracked Changes’ looks quite extensively edited.

Best regards,

Nick Van Reet
