# Supplementary material for: Single nucleotide polymorphisms and copy-number variations in the Trypanosoma brucei repeat (TBR) sequence can be used to enhance amplification and genotyping of Trypanozoon strains
Source: PLoS One. 2021 Oct 25;16(10):e0258711. doi: 10.1371/journal.pone.0258711 (PMC8544829; doi:10.1371/journal.pone.0258711)
Supplement: Attachment — Submitted filename: Response to reviewers.docx [file pone.0258711.s011.docx]

PONE-D-21-19109
Single nucleotide polymorphisms and copy-number variations in the Trypanosoma brucei repeat (TBR) sequence can be used to enhance amplification and genotyping of Trypanozoon strains
PLOS ONE

Dear Dr. Van Reet,

Thank you for submitting your manuscript to PLOS ONE. After careful consideration, we feel that it has merit but does not fully meet PLOS ONE’s publication criteria as it currently stands. Therefore, we invite you to submit a revised version of the manuscript that addresses the points raised during the review process.

Please submit your revised manuscript by 10th August 2021. If you will need more time than this to complete your revisions, please reply to this message or contact the journal office at [plosone@plos.org](mailto:plosone@plos.org). When you're ready to submit your revision, log on to <https://www.editorialmanager.com/pone/> and select the 'Submissions Needing Revision' folder to locate your manuscript file.

Please include the following items when submitting your revised manuscript:

- A rebuttal letter that responds to each point raised by the academic editor and reviewer(s). You should upload this letter as a separate file labeled 'Response to Reviewers'.
- A marked-up copy of your manuscript that highlights changes made to the original version. You should upload this as a separate file labeled 'Revised Manuscript with Track Changes'.
- An unmarked version of your revised paper without tracked changes. You should upload this as a separate file labeled 'Manuscript'.

If you would like to make changes to your financial disclosure, please include your updated statement in your cover letter. Guidelines for resubmitting your figure files are available below the reviewer comments at the end of this letter.

If applicable, we recommend that you deposit your laboratory protocols in protocols.io to enhance the reproducibility of your results. Protocols.io assigns your protocol its own identifier (DOI) so that it can be cited independently in the future. For instructions see: <http://journals.plos.org/plosone/s/submission-guidelines#loc-laboratory-protocols>. Additionally, PLOS ONE offers an option for publishing peer-reviewed Lab Protocol articles, which describe protocols hosted on protocols.io. Read more information on sharing protocols at <https://plos.org/protocols?utm_medium=editorial-email&utm_source=authorletters&utm_campaign=protocols>.

We look forward to receiving your revised manuscript.

Kind regards,

Maria Stefania Latrofa
Academic Editor
PLOS ONE

Journal Requirements:

When submitting your revision, we need you to address these additional requirements.

1. Please ensure that your manuscript meets PLOS ONE's style requirements, including those for file naming. The PLOS ONE style templates can be found at 
<https://journals.plos.org/plosone/s/file?id=wjVg/PLOSOne_formatting_sample_main_body.pdf> and 
<https://journals.plos.org/plosone/s/file?id=ba62/PLOSOne_formatting_sample_title_authors_affiliations.pdf>

2. PLOS ONE now requires that authors provide the original uncropped and unadjusted images underlying all blot or gel results reported in a submission’s figures or Supporting Information files. This policy and the journal’s other requirements for blot/gel reporting and figure preparation are described in detail at <https://journals.plos.org/plosone/s/figures#loc-blot-and-gel-reporting-requirements> and <https://journals.plos.org/plosone/s/figures#loc-preparing-figures-from-image-files>. When you submit your revised manuscript, please ensure that your figures adhere fully to these guidelines and provide the original underlying images for all blot or gel data reported in your submission. See the following link for instructions on providing the original image data: <https://journals.plos.org/plosone/s/figures#loc-original-images-for-blots-and-gels>. 

**S1_Fig already contains the full raw data of 36 electrophoresis experiments. Six of these electrophoresis experiments (the last three images from the first and the second row) were used to build Fig 2. All individual electrophoresis experiments can be provided as tiff or as .sgd file upon request.**

In your cover letter, please note whether your blot/gel image data are in Supporting Information or posted at a public data repository, provide the repository URL if relevant, and provide specific details as to which raw blot/gel images, if any, are not available. Email us at [plosone@plos.org](mailto:plosone@plos.org) if you have any questions.

Please review your reference list to ensure that it is complete and correct. If you have cited papers that have been retracted, please include the rationale for doing so in the manuscript text, or remove these references and replace them with relevant current references. Any changes to the reference list should be mentioned in the rebuttal letter that accompanies your revised manuscript. If you need to cite a retracted article, indicate the article’s retracted status in the References list and also include a citation and full reference for the retraction notice.

Reviewers' comments:

Reviewer's Responses to Questions

**Comments to the Author**

1. Is the manuscript technically sound, and do the data support the conclusions?

The manuscript must describe a technically sound piece of scientific research with data that supports the conclusions. Experiments must have been conducted rigorously, with appropriate controls, replication, and sample sizes. The conclusions must be drawn appropriately based on the data presented.

Reviewer #1: Yes

Reviewer #2: Yes

2. Has the statistical analysis been performed appropriately and rigorously?

Reviewer #1: Yes

Reviewer #2: Yes

3. Have the authors made all data underlying the findings in their manuscript fully available?

The [PLOS Data policy](http://www.plosone.org/static/policies.action#sharing) requires authors to make all data underlying the findings described in their manuscript fully available without restriction, with rare exception (please refer to the Data Availability Statement in the manuscript PDF file). The data should be provided as part of the manuscript or its supporting information, or deposited to a public repository. For example, in addition to summary statistics, the data points behind means, medians and variance measures should be available. If there are restrictions on publicly sharing data—e.g. participant privacy or use of data from a third party—those must be specified.

Reviewer #1: Yes

Reviewer #2: Yes

4. Is the manuscript presented in an intelligible fashion and written in standard English?

PLOS ONE does not copyedit accepted manuscripts, so the language in submitted articles must be clear, correct, and unambiguous. Any typographical or grammatical errors should be corrected at revision, so please note any specific errors here.

Reviewer #1: Yes

Reviewer #2: Yes

5. Review Comments to the Author

Please use the space provided to explain your answers to the questions above. You may also include additional comments for the author, including concerns about dual publication, research ethics, or publication ethics. (Please upload your review as an attachment if it exceeds 20,000 characters)

Reviewer #1: Review of Single nucleotide polymorphisms and copy-number variations in the Trypanosoma brucei repeat (TBR) sequence can be used to enhance amplification and genotyping of Trypanozoon strains by Van Reet et al.

In this paper Van Reet and co-authors analysed genetic variation in TBR sequences and developed a novel quantitative PCR molecular test (qTBR) which allows to identify Trypanozoon taxa.

The study is technically sound, and the manuscript is well written. Molecular detection of Trypanosoma is an important approach for the early diagnosis of disease. In my opinion, the manuscript represents an incremental advance that is of interest to the field.

Only minor issues should be addressed.

**Line 56 and 81-86: Update the reference (ref 1; ref 20-23)**

In line 56, we complemented the historical reference of Hoare with the more recent Radwanska *et al* 2018 “Salivarian Trypanosomosis: A Review of Parasites Involved, Their Global Distribution and Their Interaction With the Innate and Adaptive Mammalian Host Immune System”.

In line 81, we added a reference after the sentence. We updated the statement in line 84 with a reference on the genome of *Trypanosoma brucei gambiense* by Jackson *et al* 2010 “The Genome Sequence of Trypanosoma brucei gambiense, Causative Agent of Chronic Human African Trypanosomiasis”, and moved this sentence after the statement on other non-repetitive DNA which refers to *T. brucei*. In line 86, we omitted the original references 20, 21 and 22 and only retained ref 20. The original reference 23 (now reference 25) is not mentioned in lines 81-86.

This section [lines 80-87] now reads as “Around 100 MCs, sized 50-150 kb, are present in the nuclear DNA of *T. brucei* and they represent almost 10% of the nuclear genome **(19)**. It is estimated that roughly 55% of each MC, and thus 5.5% of the nuclear DNA in *T. brucei*, consists of such TBR repeats (19,20). The non-repetitive DNA on MCs carries an important part of the silent VSG gene repertoire, with most MCs having complete VSG genes that can be transposed to the VSG expression site during the early stages of an infection (**20**). In *T.b.g.,* the average lengths of the MCs are smaller, being 25 to 50 kb, and the estimated copy-numbers vary **between a few to up** to 100 (**21–24**).”

**Lines 57-59: put the HAT in 58 West and Central Africa into wider context than 2018**

We assume that the reviewer wants us to mention that *gambiense* is targeted for elimination by WHO. This section [lines 57-59] now reads as “*T.b. gambiense (T.b.g.)* is responsible for chronic human African trypanosomiasis (HAT**), a disease targeted for elimination by the World Health Organization** that **still** accounted for 977 patients reported in West and Central Africa in 2018 (3). **Annually, less than 100** cases are due to *T.b. rhodesiense (T.b.r.),* which causes **acute** HAT in East Africa (4).”

Alternatively, we could give the exact numbers of reported cases for 2020 for both gambiense and rhodesiense by referring to <https://www.who.int/data/gho/data/indicators/indicator-details/GHO/hat-tb-gambiense> and <https://www.who.int/data/gho/data/indicators/indicator-details/GHO/number-of-new-reported-cases-of-human-african-trypanosomiasis-(t-b-rhodesiense)>

**Lines 95, 96 and throughout the paper: About the possibility to assess the geographical origin of Trypanosoma strains using the TBR-PCR, more caution should be used. The future analysis of a higher number of strains would be useful to support this finding.**

We added more caution by adjusting the following statements relating to the possibility to assess the geographical origin using TBR-PCR.

Line 51, we replaced ‘it is possible to asses’ to ‘may hint at’. This section [lines 49-51] now reads as

“In addition, variations in the TBR content of Trypanozoon are apparently so large that a given qTBR profile **may hint at** the geographical origin of a given strain.”

Line 95, we replaced “asses” to “may suggest”. This section [lines 94-97] now reads as

“Furthermore, we show that single nucleotide polymorphisms and copy-number variations in the TBR sequences can be exploited to improve the amplification of all *Trypanozoon* taxa using a newly developed quantitative TBR-PCR, called qTBR, that **may** even allow to **suggest** the geographical origin of certain strains.”

Line 271, we added “present in this collection”. This section [lines 274-277] now reads as

“By plotting the target sequence RCNs obtained for q177_T_ against those obtained for q176_T_, the 77 *Trypanozoon* strains **present in this collection** are scattered along a continuum whereby both ends can be named after the geographical region from where most strains were isolated (Fig 4).“

Line 338, we added “within larger and more diverse collections of Trypanozoon” . This section [lines 340 - 342] now reads as

“With the limited TBR sequence information available today, we cannot exclude that more TBR groups may be discovered within **larger and more diverse collections** of *Trypanozoon*, perhaps even specific for certain *Trypanozoon* taxa.”

Reviewer #2: Comments to “Single nucleotide polymorphisms and copy-number variations in the Trypanosoma brucei repeat (TBR) sequence can be used to enhance amplification and genotyping of Trypanozoon strains” by Van Reet et al.

Van Reet et al proposes an interesting work on a molecular marker (TBR) used for decades in the field of Trypanosoma diagnostics. As rightly noticed by the authors, very few knowledge on this marker is available. Thus, the idea of this study is to give more insight on the molecular properties of this marker taking advantage of sequences available on the databases and of a large panel of Trypanosoma spp DNA from different geographical origin.
To do so, they first looked for TBR reads available from Tbb and Tbg database and aligned and sorted them thanks to the presence of SNPs. From this first analysis, they observed two groups of sequences: one of 177bp and the other of 176bp; both group differing from few SNPs but that cannot discriminate Tbb from Tbg. Variation of the copy number of repeats have been also detected.
Interestingly, the authors then observed that most of the current TBR primers preferentially matched with the 176bp TBR group and thus, are potentially missing the 177bp group information. They decided to design new primer sets for conventional PCR targeting both groups (calling PCR c177 and c176) and to perform semi-quantitative PCRs to compare sensitivity of these new primers with the previous ones. As suspected by in silico analysis, 177bp primer sets allowed the amplification of some Tbg1 strains that did not give PCR signal when amplified by c176 or the classical published primers.
Consequently, the authors design a multiplex qPCR with specific reverse primers for 176 and 177 (called q177T-R and q176T-R) altogether with q177T and q176T probes. They combined these 2 PCR with a 18S rDNA qPCR that will allow to normalize and calculated the relative copy-number (RCN) of each TBR target. They showed that the TBR repertoire is important within and between the different Trypanozoon taxa, with TBR RCN ranging from around 10 to more than 1000 in some cases.
To explore more deeply this variability, Van Reet et al decided to analyse the data set by plotting q177t against q176t for 77 Trypanozoon strains coming from different origins (East/Central/West Africa). Three clusters, corresponding to the three geographic origins were highlighted, independently of the Trypanosoma species.

General Comments:
Despite the complexity of the methodology used, it is clear that the authors did a great effort to make the paper as clear as possible. They go straightforward even sometimes, it makes difficult to catch the ideas from the first read. From a technical point of view, the experiments are well designed and the controls are properly used.
On one hand, the new data generated on TBR marker are interesting from a conceptual/fundamental point of view. For this, the MS deserves to be published. On the other hand, the expectation of reader is a little disappointed because it is hard to imagine than such a tool could be used to improve the diagnosis, especially to discriminate the animal-infective trypanosomes from the human-infective ones. Currently, there is an urgent need to develop new tools to do so, and the approach proposed by Van Reet et al does not fill this gap.

The reviewer is correct in stating that despite the improvements made in TBR PCR, we are not able to differentiate *T.b. gambiense* from non-*gambiense*. However, we firmly disagree that it would be hard to imagine that this PCR would improve diagnosis. Indeed, the possibility to diagnose a *Trypanozoon* infection caused by *T.b. gambiense*, is enormously improved considering the fact that previous TBR PCRs were not always able to amplify this parasite subspecies. A statement underlining this is moved higher up in the discussion to lines 314-316. **“****The dual TBR probes in the qTBR improve amplification of all Trypanozoon taxa, which is particularly relevant for trypanosomiasis infections caused by *T.b.g* I in insects and mammals (42).”**

**Specific comments:
Please define “RCN” before the “Results” section. The same for “qM”.**

Done. Both abbreviations are now defined in the Material and methods section lines 169-172.

**Figure 4: lack of Tbb from Central Africa.**

The reviewer points to a lack of *T.b.b.* strains from Central Africa. However, this is not completely the case, *T.b.b.* AnTat 17.1 was isolated from a sheep in the Democratic Republic of the Congo in 1978 and is genotyped as TBR-East. However, we agree that the absence of recently isolated *T.b.b.* strains especially from DRC, is a limitation of this study. Therefore, we added this to the limits of the study and remarks on T.b.g. I already present in the discussion. This section [lines 333-336] now reads as

“The 177 bp TBR group is detectable in all *T.b.g.* I strains **present in this collection**. **However**, more strains from **East Africa and Central Africa** ought to be included for a better geographical coverage of *T.b.g.* I (46,47). **In addition, another limit of our study is the absence of recently isolated *T.b.b.* strains from West Africa and Central Africa in our *Trypanozoon* collection.”**

**Figure 4: Improve the color code to make easier the recognition of the different species.**

We thank the reviewer for spotting this mistake. We omitted the “*T.b.g. I* MPXR” notation in Fig 4, which was a remnant of a previous version of this Figure. The updated Fig 4 still contains 8 colors (instead of 9) which cannot be further reduced. The current color scale (R-viridis) was specifically chosen to improve visualization for colorblindness and gray-scale printing. (<https://cran.r-project.org/web/packages/viridis/vignettes/intro-to-viridis.html>).

**Table 1: Find a way to help the reader to understand the primer combinations between the conventional, duplex and triplex (q)PCR. It will facilitate the reading and understanding of the MS.**

We thank the reviewer for indicating that this is indeed difficult to understand from the current section. We agree that this section in Methods describing the primers and probes of Table1 could be improved. We tried to add more clarity by describing the targets in separate sentences. The first section now reads as [lines 130-135], while in line 139 we corrected the software version of AlleleID 7.

“We used IDT PrimerQuest to design a hydrolysis probe based qPCR for the ***Trypanozoon* specific single-copy** *GPI-PLC* gene (qGPI-PLC). The conventional M18S II PCR, as described in Deborggraeve *et al* in (15), complemented with a hydrolysis probe for use in qPCR, as described by Bendofil *et al* in (41), **targets the multi-copy 18S rRNA of *Trypanozoon*** **and was abbreviated** as q18S **throughout this manuscript**. **IDT PrimerQuest was used** to design a conventional (c177) and quantitative (q177_D_) PCR based on the 80% consensus sequence of the Tbg177 set, aiming to target the 177 bp TBR group.“

In the following section we added a sentence to introduce a supplementary figure to help visualize the difference between the conventional PCR and the duplex and triplex qPCR described in Table 1. [lines 143-144] “The position of the primers and probes targeting TBR are shown in S1_Fig.” Followed by a section [lines 491-497 ] in Supporting information.

**“S1_Fig. Position of the primers and probes targeting TBR**

A representation of a TBR sequence as tandem repeat (yellow) showing the relative position of primers and probes used in conventional and quantitative PCR. Arrows indicate the 5’ – 3’ direction of primers, while for probes, circles indicate fluorophores and diamonds indicate quenchers. In A, blue indicates the c177 and the q177_D_ set, while red represents the c176 set. In B, the green arrow represents the common primer qTBR-F, while the blue primer and probe indicate the q177_T_ set, and the red primer and probe indicate the q176_T_ set. “

Finally, to further improve the interpretation of duplex and triplex qPCR, we moved the explication of the duplex reaction before the triplex qTBR reaction. This section [lines 169-176] now reads as

“In duplexed qPCR reactions, FAM-labelled probes for qGPI-PLC, q177_D_ or the qPCR described by Mumba *et al* ((30)), here abbreviated as qM, were combined with a VIC-labelled q18S probe to allow the calculation of relative copy-numbers (RCN). **These RCN, were calculated by subtracting the C_q_-values obtained in qGPI-PLC, q177_D_ or qM from the C_q_-value in q18S, resulting in a ΔC_q_-value that was transformed to 2^-ΔCq^ and averaged between replicates to yield the RCNs for each of these targets.** The qTBR reaction is performed as a triplex qPCR reaction with a NED-labelled q177_T_ probe, a FAM-labelled q176_T_ probe and a VIC-labelled q18S probe. Here, RCNs were calculated by subtracting the C_q_-values obtained in q177_T_ or q176_T_ from the C_q_-value in q18S, resulting in a ΔC_q_-value that was transformed to 2^-ΔCq^ and averaged between replicates to yield the RCNs for each TBR target”

6. PLOS authors have the option to publish the peer review history of their article ([what does this mean?](https://journals.plos.org/plosone/s/editorial-and-peer-review-process#loc-peer-review-history)). If published, this will include your full peer review and any attached files.


If you choose “no”, your identity will remain anonymous but your review may still be made public.


**Do you want your identity to be public for this peer review?** For information about this choice, including consent withdrawal, please see our [Privacy Policy](https://www.plos.org/privacy-policy).

Reviewer #1: No

Reviewer #2: No

[NOTE: If reviewer comments were submitted as an attachment file, they will be attached to this email and accessible via the submission site. Please log into your account, locate the manuscript record, and check for the action link "View Attachments". If this link does not appear, there are no attachment files.]

While revising your submission, please upload your figure files to the Preflight Analysis and Conversion Engine (PACE) digital diagnostic tool, <https://pacev2.apexcovantage.com/>. PACE helps ensure that figures meet PLOS requirements. To use PACE, you must first register as a user. Registration is free. Then, login and navigate to the UPLOAD tab, where you will find detailed instructions on how to use the tool. If you encounter any issues or have any questions when using PACE, please email PLOS at [figures@plos.org](mailto:figures@plos.org). Please note that Supporting Information files do not need this step.

*In compliance with data protection regulations, you may request that we remove your personal registration details at any time.* [*(Remove my information/details)*](https://www.editorialmanager.com/pone/login.asp?a=r)*. Please contact the publication office if you have any questions.*
